# Efficient and Accurate Separable Models for Discretized Material Optimization: A Continuous Perspective Based on Topological Derivatives

**DOI:** 10.1007/s12220-024-01663-0

**Published:** 2024-04-29

**Authors:** Peter Gangl, Nico Nees, Michael Stingl

**Affiliations:** 1 Johann Radon Institute for Computational and Applied Mathematics (RICAM), Austrian Academy of Sciences, Altenberger Straße 69, Linz, 4040 Austria; 2 Chair of Applied Mathematics (Continuous Optimization), Friedrich-Alexander-Universität Erlangen-Nürnberg, Cauerstraße 11, Erlangen, 91058 Germany

**Keywords:** Discretized material optimization, Separable models, Topological derivative, Sherman–Morrison–Woodbury formula

## Abstract

Multi-material design optimization problems can, after discretization, be solved by the iterative solution of simpler sub-problems which approximate the original problem at an expansion point to first order. In particular, models constructed from convex separable first order approximations have a long and successful tradition in the design optimization community and have led to powerful optimization tools like the prominently used method of moving asymptotes (MMA). In this paper, we introduce several new separable approximations to a model problem and examine them in terms of accuracy and fast evaluation. The models can, in general, be nonconvex and are based on the Sherman–Morrison–Woodbury matrix identity on the one hand, and on the mathematical concept of topological derivatives on the other hand. We show a surprising relation between two models originating from these two—at a first sight—very different concepts. Numerical experiments show a high level of accuracy for two of our proposed models while also their evaluation can be performed efficiently once enough data has been precomputed in an offline stage. Additionally it is demonstrated that suboptimal decisions can be avoided using our most accurate models.

## Introduction

The goal of computational design optimization is to find an optimal arrangement of possibly multiple materials inside a design region of a computational domain. Here, optimality is considered with respect to a given cost function, which most often depends on the solution of a constraining partial differential equation (PDE). Thus, a general PDE-constrained design optimization problem reads

1 $$\begin{aligned} \Omega ^* = \underset{\Omega }{\text{ arg } \text{ min } } J(\Omega , u) \quad \text{ subject } \text{ to } \quad e(\Omega ; u) = 0 \end{aligned}$$

where $e(\cdot ; \cdot )$ represents the PDE constraint and $\Omega$ can also be understood as a set of subdomains corresponding to different materials. There exist different classes of approaches to solving problems of this type. While shape optimization methods [1] can only modify existing boundaries or interfaces between subdomains in a smooth way, topology optimization approaches [2] can also alter the topology of a design and can thus admit more general solutions. In topology optimization, the design is most often represented by either a level set function [3, 4] or by means of a density function [5] that interpolates between different material properties. Note that both kinds of approaches can be extended to the case of multiple materials, see, e.g., [6, 7] or [8]. Typically, the constraining PDE is solved by a numerical method, most often by the finite element method. When approaching a design optimization problem of the type (1) by a gradient-based approach, one first has to decide whether the differentiation with respect to the design variable should be carried out before or after discretization of the problem. It should be noted that, depending on the chosen discretization and parametrization of the design, these two ways need not yield the same discrete sensitivities [9]. We mention that, of course, the numerical analysis in finite dimensions is only a part of the solution of shape and topology optimization problems. Such an analysis should be completemented by the results on the infinite dimensional model including the existence of an optimal shape and the convergence of derivative based optimization methods. Such results are obtained usually by using appropriate regularization techniques. We refer the reader, e.g., to [10].

In this paper we are interested in a (multi-material) topology optimization problem which we parametrize by a density function. Here, we focus on the approach where we first discretize the problem and then aim to solve the discretized, finite-dimensional problem. Given a computational domain ${\textsf{D}}\subset {\mathbb {R}}^2$ which is discretized using a fixed structured mesh consisting of *m* triangular elements with *n* vertices, we aim at finding the optimal material distribution ${\varvec{\lambda }}^* \in {\mathbb {R}}^m$ minimizing the heat compliance,

2 $$\begin{aligned} {\varvec{\lambda }}^* = \underset{{\varvec{\lambda }}\in \mathbb [{\underline{\lambda }}, {\overline{\lambda }}]^m}{\text{ arg } \text{ min }} \; {\mathcal {J}}({\varvec{\lambda }}) \end{aligned}$$

with ${\mathcal {J}}({\varvec{\lambda }}):= {\varvec{f}}^\top {\varvec{K}}({\varvec{\lambda }})^{-1} {\varvec{f}}$. Here, ${\varvec{K}}({\varvec{\lambda }}) \in {\mathbb {R}}^{n \times n}$ and ${\varvec{f}}\in {\mathbb {R}}^n$ represent, respectively, the (invertible) stiffness matrix and the load vector corresponding to a discretization by means of piecewise linear, globally continuous finite elements where the material coefficient in the $\ell$-th element $T_\ell$ is given by ${\varvec{\lambda }}_\ell$, $\ell \in \{1, \dots , m\}$. The problem may be subject to additional constraints, e.g., on the volume of given materials, or enriched by terms that penalize the appearance of intermediate materials or that regularize the obtained designs by filtering [11].

The idea of *sequential global programming* (SGP) [12] is the following: Instead of solving an optimization problem like (2) over $\mathbb R^m$ directly, one solves a sequence of simpler optimization problems with cost function $\hat{{\mathcal {J}}}[{\varvec{\lambda }}^{(k)}]({\varvec{\lambda }})$ which approximate the original problem with cost function $\mathcal {J}({\varvec{\lambda }})$ at an expansion point ${\varvec{\lambda }}^{(k)}$ to first order. The solution of the simpler optimization problem at iteration *k* is subsequently used as an expansion point ${\varvec{\lambda }}^{(k+1)}$ in iteration $k+1$, i.e.,

$$\begin{aligned} {\varvec{\lambda }}^{(k+1)} = \underset{{\varvec{\lambda }}\in [{\underline{\lambda }}, {\overline{\lambda }}]^m}{\text{ arg } \text{ min }} \, \hat{\mathcal {J}}[{\varvec{\lambda }}^{(k)}]({\varvec{\lambda }}). \end{aligned}$$

A class of approximate models that is of particular interest is the class of *separable* models. The use of convex, separable approximations has a long tradition in design optimization, see, e.g., [13–15] and have lead to powerful software realizations like CONLIN [16] or the method of moving asymptotes [17]. Such models allow to solve the approximate optimization problem that is posed over ${\mathbb {R}}^m$ by solving merely *m* one-dimensional optimization problems. These one-dimensional sub-problems can often be solved to global optimality. This observation holds true trivially for the more traditional convex separable approximations, used, e.g., in MMA. However, for separable approximations, convexity is not a strict requirement. It is clear that the convergence speed of an SGP algorithm strongly depends on the quality of the approximating model $\hat{{\mathcal {J}}}[{\varvec{\lambda }}^{(k)}]$. In this paper, we propose different first order separable models $\hat{{\mathcal {J}}}[{\varvec{\lambda }}^{(k)}]$ and numerically examine them in terms of accuracy and efficiency of evaluation. But it is not only the efficiency, which is of interest. This becomes particularly evident, when topology optimization or discrete material optimization problems are studied. The usual way to deal with such problems is to use a combined relaxation and penalization scheme, see, e.g., [5] for an introduction to that topic. While such approaches are very successful in practice, in extreme cases it can happen that any feasible integer solution satisfies first order optimality conditions for the continuous relaxations. Thus, there is a certain risk that rather poor local minimizers are obtained. While in literature so-called continuation strategies provide a viable concept to cope with that situation, in this paper we demonstrate that it is in particular the approximation quality in the sub-problem, which can help to avoid ’wrong’ decisions taken in the course of the iterations.

We will investigate models $\hat{{\mathcal {J}}}$ that exploit the Sherman–Morrison–Woodbury matrix identity on the discrete level and are thus purely algebraic. On the other hand, we will consider the mathematical concept of topological derivatives [18] which is a notion defined on a purely continuous setting. We will draw some, at a first glance, surprising connections between these two types of approaches and present some models that are at the same time accurate approximations of the original problem and cheap to evaluate.

The rest of this paper is organized as follows: In Sect. 2, we introduce the continuous model problem and its finite element discretization and recall the notions of topological derivatives, separable approximations of optimization problems and also the Sherman–Morrison–Woodbury formula. Next, we introduce a first efficient model based on this formula that is based on a diagonal approximation of the stiffness matrix in Sect. 3. Subsequently, we introduce a different model that is motivated by the concept of topological derivatives in Sect. 4. In Sect. 5, inspired by the procedure of the previous section, we introduce another accurate and efficient to evaluate approximation to the exact Sherman–Morrison–Woodbury model. A relation between these latter two models is established in Sect. 6. Finally, we examine all introduced models numerically in Sect. 7.

## Preliminaries

In this section, we will introduce the model problem and collect some mathematical preliminaries, which we will make use of in later sections. In particular, we introduce the mathematical concept of topological derivatives, the concept of separable first order approximations of a continuously differentiable function $f: {\mathbb {R}}^N \rightarrow {\mathbb {R}}$ and recall the Sherman–Morrison–Woodbury matrix identity.

**Notation.** Vector quantities and matrices will be denoted by bold-face symbols and we will denote the *j*-th component of a vector ${\varvec{v}}\in {\mathbb {R}}^N$ by a sub-index ${\varvec{v}}_j$. We will denote the *i*-th Cartesian unit vector in ${\mathbb {R}}^N$ by ${\varvec{e}}^{(i)}$. The identity matrix of dimension *N* will be denoted by the symbol ${\varvec{I}}_N$ and for a square matrix ${\varvec{A}}\in {\mathbb {R}}^{N\times N}$ we denote by $\text{ diag }{\varvec{A}}\in {\mathbb {R}}^{N\times N}$ the diagonal matrix corresponding to ${\varvec{A}}$, i.e., $(\text{ diag }{\varvec{A}})_{i,i} = {\varvec{A}}_{i,i}$ and $(\text{ diag }{\varvec{A}})_{i,j} = 0$ for $i\ne j$, $i, j \in \{1,\dots N\}$. We denote by $B_r(x)$ the ball of radius *r* centered at the point *x*. Moreover, given a set $A \subset \mathbb R^d$, we denote the characteristic function of the set *A* by $\chi _A(x)$, i.e., $\chi _A(x) = 1$ if $x\in A$ and $\chi _A(x) =0$ otherwise. The space of square integrable functions over a domain ${\textsf{D}}$ is denoted by $L^2({\textsf{D}})$ and the subspace of $L^2({\textsf{D}})$ functions whose weak gradient is also a $L^2({\textsf{D}})$ function is denoted by $H^1({\textsf{D}})$. Finally, given a function *f* defined on a domain ${\textsf{D}}$ and a subdomain $\Omega \subset {\textsf{D}}$, we will denote the restriction of *f* to $\Omega$ by $f|_\Omega$.

### Model Problem

As a model problem, we consider a stationary heat equation on a bounded Lipschitz domain ${\textsf{D}}\subset {\mathbb {R}}^d$. We are interested in finding the optimal material distribution within ${\textsf{D}}$ such that the heat compliance is minimized. We first state the model problem in its continuous version before introducing the discretized problem, which we are actually interested in. In this paper, we restrict ourselves to space dimensions $d=1$ and $d=2$, but remark that most concepts treated here can be extended (with some effort) also to three space dimensions.

#### Continuous Model Problem for Two Materials

For the spatial dimension $d \in \{1,2\}$, we consider the bounded Lipschitz domain ${\textsf{D}}=(0,1)^d \subset {\mathbb {R}}^d$ and a given heat source $f \in L^2({\textsf{D}})$. Given a polygonal set $\Omega \subset {\textsf{D}}$, let the piecewise constant heat conductivity $\lambda$ be defined by

3 $$\begin{aligned} \lambda _\Omega (x) = {\left\{ \begin{array}{ll} \lambda ^{\textrm{in}}, &  x \in \Omega , \\ \lambda ^{\textrm{out}}, &  x \in {\textsf{D}}\setminus {\overline{\Omega }}, \end{array}\right. } \end{aligned}$$

for two positive constants $\lambda ^{\textrm{in}}, \lambda ^{\textrm{out}}> 0$. We assume the boundary of the computational domain to be composed of a Dirichlet and a Neumann boundary, $\partial {\textsf{D}}= \Gamma _D \cup \Gamma _N$ with $\Gamma _D \cap \Gamma _N = \emptyset$, where inhomogeneous Dirichlet data $g_D$ and Neumann data $g_N$ are prescribed, respectively. We are interested in minimizing the heat compliance,

4 $$\begin{aligned} J(u) := \int \nolimits _{\textsf{D}}f(x) \, u(x) \; \text{ d }x + \int \nolimits _{\Gamma _N} g_N(x) \, u(x) \; \text{ d }s_x \end{aligned}$$

subject to a stationary heat equation. The weak formulation of the problem at hand reads

5a $$\begin{aligned}&\underset{\Omega }{\text{ inf } } J(u) \end{aligned}$$

5b $$\begin{aligned}&\text{ s.t. } u \in V_g: \; \int \nolimits _{\textsf{D}}\lambda _\Omega (x) \nabla u(x) \cdot \nabla v(x) \; \text{ d }x = \int \nolimits _{\textsf{D}}f(x) v(x) \; \text{ d }x \nonumber \\&\quad + \int \nolimits _{\Gamma _N} g_N(x) v(x) \; \text{ d }s_x \quad \forall v \in V_0 \end{aligned}$$

with the ansatz and test spaces

$$\begin{aligned} V_g := \{v \in H^1({\textsf{D}}): v|_{\Gamma _D} = g_D\}, \qquad V_0 := H^1_{\Gamma _D}({\textsf{D}}) := \{v \in H^1({\textsf{D}}): v|_{\Gamma _D} = 0\}. \end{aligned}$$

For a given subdomain $\Omega$ and assuming that $|\Gamma _D|>0$, due to the Lemma of Lax-Milgram, problem (5b) admits a unique solution which we denote by $u_\Omega$. Thus, we introduce the reduced cost function ${\mathcal {J}}(\Omega ):= J(u_\Omega )$. We assume that the solution $u_\Omega$ is sufficiently regular such that a point evaluation of its gradient $\nabla u_\Omega (z)$ is well-defined for all points $z \in D \setminus \partial \Omega$. When the set $\Omega$ is clear from the context, we will drop the index $\Omega$ and just write *u* instead of $u_\Omega$. For simplicity and without loss of generality, we assume $g_D=0$ and thus have $V_g= V_0$. The general case can be obtained by homogenization of the Dirichlet data. Note that, in the case $d=1$, the boundary of ${\textsf{D}}$ consists of two points, $\partial {\textsf{D}}= \{0,1\}$. Thus, integrals over $\Gamma _N \subset \partial {\textsf{D}}$ have to be understood as point evaluations.

The adjoint state corresponding to optimization problem (5) is the unique solution $p \in V_0$ of

6 $$\begin{aligned} \int \nolimits _{\textsf{D}}\lambda _\Omega (x) \nabla v(x) \cdot \nabla p(x) \; \text{ d }x = - \int \nolimits _{\textsf{D}}f(x) v(x) \; \text{ d }x - \int \nolimits _{\Gamma _N}g_N(x) v(x) \; \text{ d }s_x \quad \forall v \in V_0. \end{aligned}$$

Thus, it can be seen that $p = -u$.

#### Discrete Model Problem

Next, we introduce the discretization of (5) by means of piecewise linear, globally continuous finite elements. For that purpose, let ${\mathcal {T}} = \{ T_1, \dots , T_m \}$ denote a set of open simplicial elements (i.e., intervals in 1D or triangles in 2D) which form a subdivision of the computational domain ${\textsf{D}}$, i.e.,

$$\begin{aligned} {\overline{{\textsf{D}}}} = \bigcup _{\ell =1}^m {{\overline{T}}}_\ell , \qquad T_i \cap T_j = \emptyset \text{ for } i \ne j. \end{aligned}$$

Moreover, we assume that the subdomain $\Omega$ is resolved by the mesh ${\mathcal {T}}$, i.e., $\partial \Omega \cap T_\ell = \emptyset$ for all $\ell \in \{1, \dots , m\}$. Let $n \in {\mathbb {N}}$ denote the number of vertices in the mesh, $\{ \varphi _1, \dots , \varphi _n\}$ the nodal basis and $V_h:= \text {span}\{\varphi _1, \dots , \varphi _n\} \cap H^1_{\Gamma _D}({\textsf{D}})$.

Let a vector of conductivity values ${\varvec{\lambda }}\in {\mathbb {R}}^m$ be given. Note that we will sometimes identify a vector ${\varvec{\lambda }}\in {\mathbb {R}}^m$ of material values with a piecewise constant material function $\lambda (x):= \sum _{\ell =1}^m \chi _{T_\ell }(x) {\varvec{\lambda }}_\ell$. For given ${\varvec{\lambda }}\in {\mathbb {R}}^m$, the discrete version of the boundary value problem (5b) reads

7 $$\begin{aligned} {\varvec{K}}({\varvec{\lambda }}) {\varvec{u}}= {\varvec{f}}\end{aligned}$$

where the stiffness matrix ${\varvec{K}}({\varvec{\lambda }}) \in {\mathbb {R}}^{n\times n}$ and the load vector ${\varvec{f}}\in {\mathbb {R}}^n$ can be written as

8 $$\begin{aligned} {\varvec{K}}({\varvec{\lambda }}) = \sum _{\ell =1}^m {\varvec{\lambda }}_\ell {{\tilde{\varvec{B}}}_\ell }K^{(\ell )}_{loc} {{\tilde{\varvec{B}}}_\ell }^\top , \qquad {\varvec{f}}= \sum _{\ell =1}^m {{\tilde{\varvec{B}}}_\ell }f^{(\ell )}_{loc} \end{aligned}$$

with the local stiffness matrix $K^{(\ell )}_{loc} \in \mathbb R^{(d+1)\times (d+1)}$ and local load vector $f^{(\ell )}_{loc} \in {\mathbb {R}}^{d+1}$,

$$\begin{aligned} \left( K^{(\ell )}_{loc} \right) _{i,j} =&\int \nolimits _{T_\ell } \nabla \varphi _{\ell ,j} \cdot \nabla \varphi _{\ell ,i} \; \text{ d }x, \quad \quad \quad \; \quad \quad \quad i, j \in \{1, \dots , d+1\}, \\ \left( f^{(\ell )}_{loc} \right) _{i} =&\int \nolimits _{T_\ell } f \varphi _{\ell ,i} \; \text{ d }x + \int \nolimits _{\Gamma _N \cap {{\bar{T}}}_\ell } g_N \varphi _{\ell ,i} \; \text{ d }s_x, \quad i \in \{1, \dots , d+1\}, \end{aligned}$$

and the local-to-global operator ${{\tilde{\varvec{B}}}_\ell }\in {\mathbb {R}}^{n\times (d+1)}$ satisfying $({{\tilde{\varvec{B}}}_\ell })_{i,j} = 1$ if *i* is the global index of the *j*-th vertex of element $T_\ell$, and $({{\tilde{\varvec{B}}}_\ell })_{i,j} = 0$ else. Here, $\varphi _{\ell ,i}$, $i=1,\dots , d+1$, denotes the *i*-th basis functions that has non-zero support on $T_\ell$. Since we are dealing with piecewise linear and globally continuous finite elements, the local stiffness matrix can be written as

9 $$\begin{aligned} K^{(\ell )}_{loc} = {\varvec{D}}_\ell {\varvec{D}}_\ell ^\top \end{aligned}$$

with some constant matrices ${\varvec{D}}_\ell \in {\mathbb {R}}^{(d+1)\times d}$ depending solely on the coordinates of the vertices of element $T_\ell$. Thus, defining ${\varvec{B}_{\ell }}:= {{\tilde{\varvec{B}}}_\ell }{\varvec{D}}_\ell \in {\mathbb {R}}^{n \times d}$, the stiffness matrix can also be written as

10 $$\begin{aligned} {\varvec{K}}({\varvec{\lambda }}) = \sum _{\ell =1}^m {\varvec{\lambda }}_\ell {\varvec{B}_{\ell }}{\varvec{B}_{\ell }}^\top . \end{aligned}$$

##### Remark 1

In dimension $d=1$, the matrix ${\varvec{D}}_\ell \in {\mathbb {R}}^{2\times 1}$ corresponding to an element $T_\ell = (x_{\ell -1}, x_{\ell })$ is given by

$$\begin{aligned} {\varvec{D}}_\ell = \frac{1}{\sqrt{x_{\ell } - x_{\ell -1}}} \begin{pmatrix} -1 \\ 1 \end{pmatrix}. \end{aligned}$$

For $d=2$ and a triangular element $T_\ell$ with vertices ${\varvec{x}}_{\ell ,1}, {\varvec{x}}_{\ell ,2}, {\varvec{x}}_{\ell ,3}$ in counter-clockwise enumeration, the matrix ${\varvec{D}}_\ell \in {\mathbb {R}}^{3 \times 2}$ reads

$$\begin{aligned} {\varvec{D}}_\ell = \sqrt{ \frac{1}{2} \text{ det }{\varvec{J}}_\ell } \begin{pmatrix} -1 &  -1 \\ 1 &  0 \\ 0 &  1 \end{pmatrix} {\varvec{J}}_\ell ^{-1} \end{aligned}$$

with $${\varvec{J}}_\ell = \begin{pmatrix} {\varvec{x}}_{\ell ,2} - {\varvec{x}}_{\ell ,1}&{\varvec{x}}_{\ell ,3} - {\varvec{x}}_{\ell ,1} \end{pmatrix} \in {\mathbb {R}}^{2 \times 2}$$.

Finally, the Dirichlet boundary conditions on nodes ${\varvec{v}}^{(i)}$ on $\overline{\Gamma }_D$ are incorporated by setting $({\varvec{K}}({\varvec{\lambda }}))_{i,i} = 1$ and $({\varvec{K}}({\varvec{\lambda }}))_{i,j} = ({\varvec{K}}({\varvec{\lambda }}))_{j,i} = 0$ for $i \ne j$ and ${\varvec{f}}_i = g_D({\varvec{v}}_i)$. Note that, for ${\varvec{\lambda }}\in [{\underline{\lambda }}, {\overline{\lambda }}]^m$ with ${\underline{\lambda }}>0$, the stiffness matrix after incorporation of the Dirichlet boundary conditions is invertible. Thus, we can define the solution vector

11 $$\begin{aligned} {\varvec{u}}({\varvec{\lambda }}) := {\varvec{K}}({\varvec{\lambda }})^{-1} {\varvec{f}}\end{aligned}$$

and the corresponding discrete solution $u_h(x):= \sum _{i=1}^n {\varvec{u}}_i \varphi _i(x)$, and we introduce the discrete compliance function ${\mathcal {J}}: [{\underline{\lambda }}, {\overline{\lambda }}]^m \rightarrow {\mathbb {R}}$,

12 $$\begin{aligned} {\mathcal {J}}({\varvec{\lambda }}) := {\varvec{f}}^\top {\varvec{K}}({\varvec{\lambda }})^{-1} {\varvec{f}}. \end{aligned}$$

##### Remark 2

In order to obtain practically interesting multi-material designs, the discretized problem should additionally include a mechanism to penalize intermediate material. This can be done by adding a term of the form $J_{\text {gray}}({\varvec{\lambda }}) = \sum _\ell |T_\ell | ({\varvec{\lambda }}_\ell - \lambda ^{\textrm{in}})(\lambda ^{\textrm{out}}- {\varvec{\lambda }}_\ell )$ (or an extension of this to multiple materials) to the cost function. On the other hand, it is well-known that topology optimization problems of the type (2) often lack a solution which can be observed numerically in the form of mesh-dependent optimized designs. In order to obtain a well-defined problem, one typically introduces a length scale in the form of a filter radius. This can be realized by adding a term of the form $J_{\text {reg}}({\varvec{\lambda }}) = \Vert {\textbf{F}}_R {\varvec{\lambda }}- {\varvec{\lambda }}\Vert _2^2$ to the cost function. Here, ${\textbf{F}}_R$ is a filtering operator with a given length scale *R*. Thus, one typically is interested in minimizing an enriched cost function $\tilde{{\mathcal {J}}}({\varvec{\lambda }}) = {\mathcal {J}}({\varvec{\lambda }}) + \gamma _1 J_{\text {gray}}({\varvec{\lambda }}) + \gamma _2 J_{\text {reg}}({\varvec{\lambda }})$. Since the functionals $J_{\text {gray}}({\varvec{\lambda }})$ and $J_{\text {reg}}({\varvec{\lambda }})$ are often separable and can be evaluated efficiently by default, we will ignore these terms for the rest of this paper. For a more detailed discussion on this aspect for multi-material topology optimization, see [19, Sec. 2.2].

Later on, we will make use of the following relation.

##### Lemma 1

Let $u_h$ a piecewise linear and globally continuous finite element function on a given simplicial mesh in ${\mathbb {R}}^d$, $d \in \{1,2\}$ with vector of basis coefficients ${\varvec{u}}\in {\mathbb {R}}^n$ and let ${\varvec{B}_{\ell }}= {{\tilde{\varvec{B}}}_\ell }{\varvec{D}}_\ell$ defined as above. Then it holds for any $\ell \in \{1,\dots , m\}$

13 $$\begin{aligned} {\varvec{B}_{\ell }}^\top {\varvec{u}}= \sqrt{|T_\ell |} \, \nabla u_h|_{T_\ell }. \end{aligned}$$

##### Proof

First note that ${\varvec{u}}^\top {\varvec{B}_{\ell }}= {\varvec{u}}^\top {{\tilde{\varvec{B}}}_\ell }{\varvec{D}}_\ell$ and that ${\varvec{u}}^\top {{\tilde{\varvec{B}}}_\ell }$ is the vector of local degrees of freedom on element $T_\ell$. Thus, for $d=1$, we get ${\varvec{u}}^\top {\varvec{B}_{\ell }}= [{\varvec{u}}_{\ell ,1}, {\varvec{u}}_{\ell ,2}] {\varvec{D}}_\ell = \sqrt{|T_\ell |}({\varvec{u}}_{\ell ,2} - {\varvec{u}}_{\ell ,1})/|T_\ell |$. The assertion follows by recalling that $|T_\ell |$ is the length of the interval $T_\ell$ and that $u_h$ is linear on $T_\ell$.

In order to see the relation in the case $d=2$, let $\Phi _\ell$ denote the affine transformation with Jacobian matrix ${\varvec{J}}_\ell$ that maps the reference triangle with vertices $(0,0)^\top$, $(1,0)^\top$, $(0,1)^\top$ to the given physical triangle $T_\ell$. Recall that, by the chain rule $(\nabla u_h)\circ \Phi _\ell = {\varvec{J}}_\ell ^{-\top } \nabla {{\hat{u}}}$ with ${{\hat{u}}} =u_h\circ \Phi _\ell$ and thus $\nabla {{\hat{u}}}^\top {\varvec{J}}_\ell ^{-1} = ((\nabla u_h)\circ \Phi _\ell )^\top$. Now we have

$$\begin{aligned} {\varvec{u}}^\top {\varvec{B}_{\ell }}= [{\varvec{u}}_{\ell ,1}, {\varvec{u}}_{\ell ,2}, {\varvec{u}}_{\ell ,3}] {\varvec{D}}_\ell = \sqrt{ \frac{1}{2} \text{ det }{\varvec{J}}_\ell } [{\varvec{u}}_{\ell ,2}-{\varvec{u}}_{\ell ,1},{\varvec{u}}_{\ell ,3}-{\varvec{u}}_{\ell ,1} ] {\varvec{J}}_\ell ^{-1}, \end{aligned}$$

and noting that $[{\varvec{u}}_{\ell ,2}-{\varvec{u}}_{\ell ,1},{\varvec{u}}_{\ell ,3}-{\varvec{u}}_{\ell ,1} ] = \nabla {{\hat{u}}}^\top$ and $\text{ det }{\varvec{J}}_\ell = 2 |T_\ell |$ finishes the proof. $\square$

***Chosen meshes.***

**Fig. 1:**
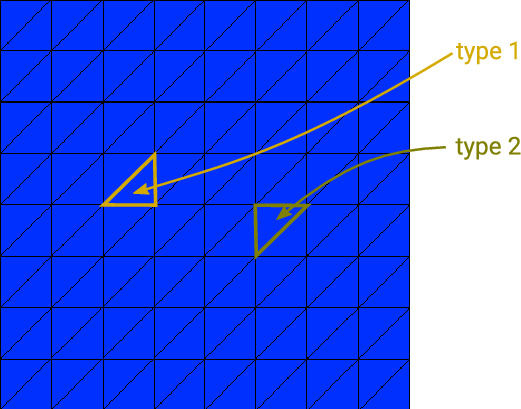
Example of structured mesh as used in this paper consisting of only two different types of elements

Given a refinement level $n_{\text {ref}} \in \{4,5,6\}$, we use a structured mesh with $2^{n_{\text {ref}}}+1$ many uniformly distributed points per dimension. For $d=1$ this corresponds to a uniform grid with $n=2^{n_{\text {ref}}}+1$ points and $m= 2^{n_{\text {ref}}}$ elements. For $d=2$, we have $n = (2^{n_{\text {ref}}}+1)^2$ and $m= 2^{2n_{\text {ref}}+1}$ triangular elements. The triangles are obtained by dividing each square in the Cartesian grid created by the vertices into two triangles with a diagonal connecting the bottom left and top right vertex of a square, see Fig. 1. As a result, our triangular mesh contains only two types of triangles (both being isosceles right triangles): Element type 1 having the right angle on the bottom right, and element type 2 having the right angle on the top left, see Fig. 1.

### Topological Derivative

The topological derivative of a shape function ${\mathcal {J}} = {\mathcal {J}}(\Omega )$ represents the sensitivity of ${\mathcal {J}}$ with respect to a local topological perturbation of the domain $\Omega$ around an inner point *z*. Consider the setting introduced in Sect. 2.1.1 where $\Omega$ denotes a subdomain of a domain ${\textsf{D}}$. Let $\omega \subset {\mathbb {R}}^d$ with ${\textbf{0}} \in \omega$ represent the shape of the considered perturbation, e.g., $\omega = B_1({\textbf{0}})$ the unit ball for circular inclusion shapes, and let $z \in \Omega \cup ({\textsf{D}}{\setminus } \overline{\Omega })$. For $\varepsilon > 0$, we define the perturbation of shape $\omega$ and size $\varepsilon$ as $\omega _\varepsilon (z):= z + \varepsilon \omega$.

#### Definition 1

(**topological derivative**) The topological derivative of a shape function ${\mathcal {J}}$ at the point $z\in \Omega \cup ({\textsf{D}}\setminus {\overline{\Omega }})$ with respect to the inclusion shape $\omega$ is defined by

14 $$\begin{aligned} d {\mathcal {J}}(\Omega )(z, \omega ) := {\left\{ \begin{array}{ll} \underset{\varepsilon \searrow 0}{\text{ lim } } \frac{1}{|\omega _\varepsilon |} ({\mathcal {J}}(\Omega \setminus {\overline{\omega }}_\varepsilon ) - {\mathcal {J}}(\Omega ) ), &  z \in \Omega , \\ \underset{\varepsilon \searrow 0}{\text{ lim } } \frac{1}{|\omega _\varepsilon |} ({\mathcal {J}}(\Omega \cup \omega _\varepsilon ) - {\mathcal {J}}(\Omega ) ), &  z \in {\textsf{D}}\setminus {\overline{\Omega }}. \end{array}\right. } \end{aligned}$$

#### Remark 3

Note that Definition 1 is equivalent to stating that $d{\mathcal {J}}(\Omega )(z,\omega )$ is the first term in a topological asymptotic expansion of the form (here for $z \in {\textsf{D}}{\setminus } {\overline{\Omega }}$)

15 $$\begin{aligned} {\mathcal {J}}(\Omega \cup \omega _\varepsilon ) = {\mathcal {J}}(\Omega ) + |\omega _\varepsilon | d {\mathcal {J}}(\Omega )(z, \omega ) + o(|\omega _\varepsilon |). \end{aligned}$$

In general, the topological derivative of PDE-constrained topology optimization problems with elliptic PDE constraints where the principal part of the PDE operator is perturbed involves the solution to an exterior corrector equation, which we define in the following. We refer the reader to [20] for a comprehensive introduction to the numerical computation of topological derivatives for arbitrary inclusion shapes.

#### Definition 2

Let $\omega \subset {\mathbb {R}}^d$ open with $0 \in \omega$ and let

16 $$\begin{aligned} \lambda _\omega ^{i \rightarrow j}(x) = \chi _{\omega }(x) \lambda ^j + \chi _{{\mathbb {R}}^d \setminus {\overline{\omega }}}(x) \lambda ^i \end{aligned}$$

for $i, j \in \{\text {in},\text {out}\}$, $i\ne j$. Furthermore, for $z \in \Omega \cup ({\textsf{D}}\setminus {\overline{\Omega }})$, let $\nabla u(z)$ denote the point evaluation of the gradient of the solution *u* to (5b) at *z*. For any given $\zeta \in \mathbb R^d$, we define the corrector function $K_\omega [\lambda ^i, \lambda ^j;\zeta ] \in {\dot{BL}}({\mathbb {R}}^d)$ for switching from material $\lambda ^i$ to $\lambda ^j$, $i, j \in \{\text {in},\text {out}\}$, $i\ne j$, as the unique solution to

17 $$\begin{aligned} \int \nolimits _{{\mathbb {R}}^d} \lambda _\omega ^{i \rightarrow j} \nabla K_\omega [\lambda ^i, \lambda ^j;\zeta ](x) \cdot \nabla v(x) \; \text{ d }x = -(\lambda ^j - \lambda ^i) \int \nolimits _\omega \zeta \cdot \nabla v(x) \; \text{ d }x \end{aligned}$$

for all $$v \in BL({\mathbb {R}}^d)$$.

Here, $BL({\mathbb {R}}^d):= \{v \in H^1_{\text {loc}}({\mathbb {R}}^d): \nabla v \in L^2({\mathbb {R}}^d)^d \}$ denotes the so-called Beppo-Levi space of locally square integrable functions whose gradient is square integrable over the whole unbounded domain and ${\dot{BL}}({\mathbb {R}}^d):= BL({\mathbb {R}}^d)/{\mathbb {R}}$ is the space of equivalence classes where the constants are factored out, see also [21, 22].

#### Remark 4

Note that, for $i, j \in \{\text {in},\text {out}\}$, $i\ne j$, the mapping $\zeta \mapsto K_\omega [\lambda ^i, \lambda ^j;\zeta ]$ is linear and we have for $d=2$

$$\begin{aligned} K_\omega [\lambda ^i, \lambda ^j;\nabla u(z)] = \frac{\partial u}{\partial x_1}(z) K_\omega [\lambda ^i, \lambda ^j;{\varvec{e}}^{(1)}] + \frac{\partial u}{\partial x_2}(z) K_\omega [\lambda ^i, \lambda ^j;{\varvec{e}}^{(2)}]. \end{aligned}$$

#### Proposition 2

Let $\omega \in {\mathbb {R}}^d$ open with $0 \in \omega$. Let further *p* be the adjoint state defined in (6) and $K_\omega [\lambda ^{\textrm{in}}, \lambda ^{\textrm{out}};\cdot ]$, $K_\omega [\lambda ^{\textrm{out}}, \lambda ^{\textrm{in}};\cdot ]$ according to Definition 2. The topological derivative of problem (5) with respect to $\omega$ for $z \in {\textsf{D}}{\setminus } {\overline{\Omega }}$ reads

18 $$\begin{aligned}&d {\mathcal {J}}[\lambda ^{\textrm{out}}, \lambda ^{\textrm{in}}](\Omega )(z, \omega ) \nonumber \\&\quad = (\lambda ^{\textrm{in}}- \lambda ^{\textrm{out}}) \frac{1}{|\omega |} \int \nolimits _\omega ( \nabla u(z) + \nabla K_\omega [\lambda ^{\textrm{out}}, \lambda ^{\textrm{in}};\nabla u(z)](x)) \cdot \nabla p(z) \; \text{ d }x. \end{aligned}$$

Likewise, for $z \in \Omega$, the topological derivative is given by

19 $$\begin{aligned}&d {\mathcal {J}}[\lambda ^{\textrm{in}}, \lambda ^{\textrm{out}}](\Omega )(z, \omega ) \nonumber \\&\quad = (\lambda ^{\textrm{out}}- \lambda ^{\textrm{in}}) \frac{1}{|\omega |} \int \nolimits _\omega ( \nabla u(z) + \nabla K_\omega [\lambda ^{\textrm{in}}, \lambda ^{\textrm{out}};\nabla u(z)](x)) \cdot \nabla p(z) \; \text{ d }x. \end{aligned}$$

#### Proof

For a detailed proof, see, e.g., [22]. Moreover, the idea of the proof is outlined in Sect. 4.1 where the focus is on triangular inclusion shapes. $\square$

#### Definition 3

(*weak polarization matrix*) For $\omega \in {\mathbb {R}}^d$ open with $0 \in \omega$, $\zeta \in {\mathbb {R}}^d$ and $i,j\in \{\text {in}, \text {out}\}, i\ne j$, let $K_\omega [\lambda ^i, \lambda ^j; \zeta ]$ be as defined in Definition 2. We introduce the weak polarization matrix

20 $$\begin{aligned} {\mathcal {P}}_\omega [\lambda ^i, \lambda ^j] = \left[ \frac{1}{|\omega |} \int \nolimits _\omega \nabla K_\omega [\lambda ^i, \lambda ^j; {\varvec{e}}^{(1)}]\text{ d }x \quad \frac{1}{|\omega |} \int \nolimits _\omega \nabla K_\omega [\lambda ^i, \lambda ^j; {\varvec{e}}^{(2)}]\text{ d }x \right] \in {\mathbb {R}}^{d\times d}. \end{aligned}$$

Using (20) and Remark 4, in the case $z \in {\textsf{D}}\setminus {\overline{\Omega }}$, we can also write (18) as

21 $$\begin{aligned} d {\mathcal {J}}[\lambda ^{\textrm{out}}, \lambda ^{\textrm{in}}](\Omega )(z, \omega ) = (\lambda ^{\textrm{in}}- \lambda ^{\textrm{out}}) \nabla u(z)^\top ({\varvec{I}}_2 + {\mathcal {P}}_\omega [\lambda ^{\textrm{out}}, \lambda ^{\textrm{in}}]) \nabla p(z), \end{aligned}$$

and an analogous formula is obtained for the case $z \in \Omega$. For a detailed introduction to the concept of polarization tensors, we refer the reader to the book [23].

It can be seen that the evaluation of the topological derivative at a single point *z* involves the solution of problem (17) with $\zeta = \nabla u(z)$. In the special cases where $\omega$ is an interval ($d=1$), a disk or ellipse ($d=2$) or a ball or ellipsoid ($d=3$), the solution to problem (17) can be written explicitly in a closed form. For $d \in \{2,3\}$ and $\omega = B_1(0)$ we have $\nabla K_\omega [\lambda ^i, \lambda ^j; \nabla u(z)]|_\omega = -(\lambda ^j - \lambda ^i)/(\lambda ^j + (d-1)\lambda ^i) \nabla u(z)$ and thus ${\mathcal {P}}_\omega [\lambda ^i, \lambda ^j] = -(\lambda ^j - \lambda ^i)/(\lambda ^j + (d-1)\lambda ^i) {\varvec{I}}_d$ and [24]

22 $$\begin{aligned} d {\mathcal {J}}[\lambda ^i, \lambda ^j](\Omega )(z, B_1(0)) = d \lambda ^i \frac{\lambda ^j - \lambda ^i}{\lambda ^j + (d-1)\lambda ^i} \nabla u(z) \cdot \nabla p (z). \end{aligned}$$

For $d=1$ and $\omega = (-1,1)$ we have ${\mathcal {P}}_\omega [\lambda ^i, \lambda ^j] = -(\lambda ^j-\lambda ^i)/\lambda ^j \in {\mathbb {R}}$ and thus [25]

$$\begin{aligned} d {\mathcal {J}}[\lambda ^i, \lambda ^j](\Omega )(z, B_1(0)) = \frac{\lambda ^i}{\lambda ^j}(\lambda ^j - \lambda ^i) u'(z) p'(z). \end{aligned}$$

#### Remark 5

For a general inclusion shape $\omega$, (17) cannot be solved analytically. The same holds true for the case of quasilinear PDE constraints where the problem corresponding to (17) is quasilinear as well. In these cases, however, it is still possible to get a good approximation to the topological derivative values (18), (19) by computing a numerical approximation of the solution of (17) on a comparably large, but bounded domain $B_R(0) \supset \omega$ (e.g. $R=30$) with homogeneous Dirichlet boundary conditions at $\partial B_R(0)$. This procedure is motivated by the fact that the solution $K^{i\rightarrow j}_\omega$ to (17) often can be shown to decay as $|x| \rightarrow \infty$. We refer the reader to [20] for a detailed discussion of this aspect. We will also follow this approach in Sect. 4 for triangular shaped inclusions $\omega$.

Note that, when no closed form solution is available, in the case of linear PDE constraints with only two different materials $\lambda ^{\textrm{in}}$, $\lambda ^{\textrm{out}}$ it is sufficient to have access to (an approximation of) $K_\omega [\lambda ^{\textrm{in}}, \lambda ^{\textrm{out}}; {\varvec{e}}^{(k)}]$ and $K_\omega [\lambda ^{\textrm{out}}, \lambda ^{\textrm{in}}; {\varvec{e}}^{(k)}]$ for $k=1, \dots , d$ in order to (approximately) evaluate the topological derivative in the full domain in an efficient way. Thus, problem (17) has to be solved numerically only 2*d* many times in a pre-computation stage. For the case of quasilinear PDE constraints this precomputation stage is more involved, see [26, 27].

### Separable Approximations

Problem (2) can be solved efficiently by the idea of sequential global programming (SGP) [12] where the original optimization problem is replaced by a sequence of simpler sub-problems. For these sub-problems it is beneficial to have approximations of the original objective functions, which are separable, since then solving the sub-problem reduces to the solution of several univariate optimization problems. The following definitions can also be found in [19].

#### Definition 4

(*separable function*) Let $N \in {\mathbb {N}}$. A function $g: {\mathbb {R}}^N \rightarrow {\mathbb {R}}$ is called *separable* if there exist functions $g_1, \dots , g_N: {\mathbb {R}} \rightarrow {\mathbb {R}}$ and a constant $g_0 \in {\mathbb {R}}$ such that for all ${\varvec{x}}\in {\mathbb {R}}^N$

$$\begin{aligned} g({\varvec{x}}) = g_0 + \sum _{i=1}^N g_i({\varvec{x}}_i). \end{aligned}$$

We call a model *g* exact if it still coincides with the original function *f* when only one component is perturbed.

#### Definition 5

(*separable exact model*) Let $N \in {\mathbb {N}}$, ${\overline{{\varvec{x}}}} \in {\mathbb {R}}^N$ and $f: {\mathbb {R}}^N \rightarrow {\mathbb {R}}$ be given. A separable function $g: {\mathbb {R}}^N \rightarrow {\mathbb {R}}$ is called a *separable exact model* of *f* at ${\overline{{\varvec{x}}}}$ if

23 $$\begin{aligned} g({\overline{{\varvec{x}}}} + \delta x \, {\varvec{e}}^{(i)}) = f({\overline{{\varvec{x}}}} + \delta x \, {\varvec{e}}^{(i)}) \end{aligned}$$

for all $i \in \{1, \dots N\}$ and all $\delta x \in {\mathbb {R}}$.

#### Definition 6

(*separable first order approximation*) Let $N \in {\mathbb {N}}$, ${\mathcal {I}} \subset {\mathbb {R}}^N$, $\overline{{\varvec{x}}}\in {\mathcal {I}}$ and $f \in C^1({\mathcal {I}}, {\mathbb {R}})$ be given. A function $g \in C^1({\mathcal {I}}, {\mathbb {R}})$ is called a *separable first order approximation* of *f* at $\overline{{\varvec{x}}}$ if *g* is separable and

24 $$\begin{aligned} f({\overline{{\varvec{x}}}}) = g({\overline{{\varvec{x}}}}) \qquad \text{ and } \qquad \nabla f({\overline{{\varvec{x}}}}) = \nabla g({\overline{{\varvec{x}}}}). \end{aligned}$$

Note that if a function *g* is a separable exact model of a function *f* it is also a separable first order approximation. In the following lemma we show how, for any given function *f*, a separable exact model can be constructed.

#### Lemma 3

Let $N \in {\mathbb {N}}$, ${\overline{{\varvec{x}}}} \in {\mathbb {R}}^N$ and $f: {\mathbb {R}}^N \rightarrow {\mathbb {R}}$ be given. For $j \in \{1, \dots , N\}$ define

25 $$\begin{aligned} g_j({\overline{{\varvec{x}}}}; \cdot ) : {\mathbb {R}} \rightarrow {\mathbb {R}}, \qquad g_j({\overline{{\varvec{x}}}};s) = f({\overline{{\varvec{x}}}} + (s - {\overline{{\varvec{x}}}}_j) {\varvec{e}}^{(j)}) - f({\overline{{\varvec{x}}}}). \end{aligned}$$

Then, *g* defined by

26 $$\begin{aligned} g : {\mathbb {R}}^N \rightarrow {\mathbb {R}}, \qquad g({\varvec{x}}) = f(\overline{{\varvec{x}}}) + \sum _{j=1}^N g_j({\overline{{\varvec{x}}}};{\varvec{x}}_j) \end{aligned}$$

is a separable exact model of *f* at ${\overline{{\varvec{x}}}}$.

#### Proof

It is obvious from (26) that *g* is separable. In order to see (23), let $i \in \{1, \dots , N\}$ be fixed. Noting that

$$\begin{aligned} ({\overline{{\varvec{x}}}} + \delta x \, {\varvec{e}}^{(i)})_j ={\left\{ \begin{array}{ll} \overline{{\varvec{x}}}_i + \delta x, &  j=i, \\ {\overline{{\varvec{x}}}}_j, &  j \in \{1,\dots ,N\} \setminus \{i\} , \end{array}\right. } \end{aligned}$$

we have

$$\begin{aligned} g({\overline{{\varvec{x}}}} + \delta x \,{\varvec{e}}^{(i)}) =&f({\overline{{\varvec{x}}}}) + \sum _{j=1}^N g_j({\overline{{\varvec{x}}}}; ({\overline{{\varvec{x}}}} + \delta x \,{\varvec{e}}^{(i)})_j) \\ =&f({\overline{{\varvec{x}}}}) + g_i({\overline{{\varvec{x}}}}; {\overline{{\varvec{x}}}}_i + \delta x) + \sum _{j \ne i} g_j({\overline{{\varvec{x}}}}; {\overline{{\varvec{x}}}}_j) \\ =&f({\overline{{\varvec{x}}}}) + f({\overline{{\varvec{x}}}} + \delta x \,{\varvec{e}}^{(i)}) - f({\overline{{\varvec{x}}}}) = f({\overline{{\varvec{x}}}} + \delta x \,{\varvec{e}}^{(i)}), \end{aligned}$$

where we used $g_j({\overline{{\varvec{x}}}}; {\overline{{\varvec{x}}}}_j) = 0$. This finishes the proof. $\square$

While Lemma 3 defines a separable exact model *g* for any function *f*, it should be noted that the evaluation of *g* involves $N+1$ function evaluations of *f*, which can in practice be prohibitively expensive (in particular when a function evaluation involves the solution of a PDE). Thus, considering the problem at hand (2), our goal in this paper is to find close approximations of the separable exact model defined by (26) which are cheap to evaluate.

### The Sherman–Morrison–Woodbury Formula in a Finite Element Context

The following linear algebra result will prove useful for defining exact models in the context of discretized PDE-constrained material optimization. It gives a formula for the inverse of a perturbed matrix only in terms of the inverse of the unperturbed matrix.

#### Lemma 4

(Sherman–Morrison–Woodbury formula [28]) Let $N, k \in {\mathbb {N}}$ and matrices ${\varvec{A}}\in {\mathbb {R}}^{N \times N}$ invertible, ${\varvec{U}}\in {\mathbb {R}}^{N \times k}$, ${\varvec{V}}\in \mathbb R^{k \times N}$ be given. It holds

27 $$\begin{aligned} ({\varvec{A}}+{\varvec{U}}{\varvec{V}})^{-1} = {\varvec{A}}^{-1} - {\varvec{A}}^{-1} {\varvec{U}}({\varvec{I}}_k + {\varvec{V}}{\varvec{A}}^{-1} {\varvec{U}})^{-1} {\varvec{V}}{\varvec{A}}^{-1}. \end{aligned}$$

Lemma 4 gives rise to a separable exact model of the discrete compliance functional ${\mathcal {J}} = {\mathcal {J}}({\varvec{\lambda }})$ defined in (12). For that purpose, let the material distribution ${\varvec{\lambda }}\in [{\underline{\lambda }}, {\overline{\lambda }}]^m \in {\mathbb {R}}^m$ be given and fix an element $T_\ell$, $\ell \in \{1,\dots ,m\}$. We consider a perturbation of ${\varvec{\lambda }}$ in this element and define the perturbed vector ${\varvec{\eta }}:= {\varvec{\lambda }}+ (\eta - {\varvec{\lambda }}_\ell ) {\varvec{e}}^{(\ell )}$ for some $\eta \in [{\underline{\lambda }}, {\overline{\lambda }}]$. Note that ${\varvec{\eta }}$ coincides with ${\varvec{\lambda }}$ in all components except for component $\ell$ where it has value $\eta$ rather than ${\varvec{\lambda }}_\ell$. Recall the definition of the matrices ${\varvec{B}_{\ell }}$, $\ell \in \{1,\dots , m\}$ from Sect. 2.1.2.

#### Proposition 5

Let ${\varvec{\lambda }}\in {\mathbb {R}}^m$, $\ell \in \{1,\dots , m\}$ fixed and ${\varvec{\eta }}:= {\varvec{\lambda }}+ (\eta - {\varvec{\lambda }}_{\ell }) {\varvec{e}}^{(\ell )}$. Then

28 $$\begin{aligned} {\mathcal {J}}({\varvec{\eta }}) = {\mathcal {J}}({\varvec{\lambda }}) - |T_\ell | (\eta - {\varvec{\lambda }}_{\ell }) (\nabla u_h|_{T_\ell })^\top ( {\varvec{I}}_d + (\eta - {\varvec{\lambda }}_{\ell }){\varvec{B}_{\ell }}^\top {\varvec{K}}({\varvec{\lambda }})^{-1} {\varvec{B}_{\ell }})^{-1} \nabla u_h|_{T_\ell }. \end{aligned}$$

#### Proof

The stiffness matrices according to ${\varvec{\lambda }}$ and ${\varvec{\eta }}$ read

29 $$\begin{aligned} {\varvec{K}}({\varvec{\lambda }}) = \sum _{k = 1}^m {\varvec{\lambda }}_k {B}_k {B}_k^\top \quad \text{ and } \quad {\varvec{K}}({\varvec{\eta }}) = \sum _{k = 1}^m {\varvec{\eta }}_k {B}_k {B}_k^\top = {\varvec{K}}({\varvec{\lambda }}) + (\eta -{\varvec{\lambda }}_{\ell }) {\varvec{B}_{\ell }}{\varvec{B}_{\ell }}^\top . \end{aligned}$$

Thus, the inverse of ${\varvec{K}}({\varvec{\eta }})$ can be obtained by means of Lemma 4 by setting ${\varvec{A}}= {\varvec{K}}({\varvec{\lambda }}) \in {\mathbb {R}}^{n\times n}$, ${\varvec{U}}= (\eta -{\varvec{\lambda }}_{\ell }){\varvec{B}_{\ell }}\in {\mathbb {R}}^{n\times d}$ and ${\varvec{V}}= {\varvec{B}_{\ell }}^\top \in {\mathbb {R}}^{d\times n}$ as

30 $$\begin{aligned} {\varvec{K}}({\varvec{\eta }})^{-1} {=} {\varvec{K}}({\varvec{\lambda }})^{-1} {-} (\eta {-} {\varvec{\lambda }}_{\ell }){\varvec{K}}({\varvec{\lambda }})^{-1} {\varvec{B}_{\ell }}( {\varvec{I}}_d {+} (\eta {-} {\varvec{\lambda }}_{\ell }){\varvec{B}_{\ell }}^\top {\varvec{K}}({\varvec{\lambda }})^{-1} {\varvec{B}_{\ell }})^{-1} {\varvec{B}_{\ell }}^\top {\varvec{K}}({\varvec{\lambda }})^{-1} . \end{aligned}$$

Thus, defining ${{\tilde{\varvec{u}}}}= {\varvec{K}}({\varvec{\eta }})^{-1}{\varvec{f}}$ and using that ${\varvec{u}}= {\varvec{K}}({\varvec{\lambda }})^{-1}{\varvec{f}}$ and ${\varvec{K}}({\varvec{\lambda }}) = {\varvec{K}}({\varvec{\lambda }})^\top$, it holds

31 $$\begin{aligned} {\varvec{f}}^\top {{\tilde{\varvec{u}}}}= {\varvec{f}}^\top {\varvec{u}}- (\eta - {\varvec{\lambda }}_{\ell }) {\varvec{u}}^\top {\varvec{B}_{\ell }}( {\varvec{I}}_d + (\eta - {\varvec{\lambda }}_{\ell }){\varvec{B}_{\ell }}^\top {\varvec{K}}({\varvec{\lambda }})^{-1} {\varvec{B}_{\ell }})^{-1} {\varvec{B}_{\ell }}^\top {\varvec{u}}. \end{aligned}$$

Noting that ${\mathcal {J}}({\varvec{\eta }}) = {\varvec{f}}^\top {{\tilde{\varvec{u}}}}$, ${\mathcal {J}}({\varvec{\lambda }}) = {\varvec{f}}^\top {\varvec{u}}$ and, by Lemma 1, ${\varvec{B}_{\ell }}^\top {\varvec{u}}= \sqrt{|T_\ell |} \nabla u_h|_{T_\ell }$ finishes the proof. $\square$

#### Remark 6

We remark that this procedure can also be followed for non-selfadjoint problems. Using the adjoint state $p_h \in V_h$ whose basis vector ${\varvec{p}}$ is obtained as the solution of ${{\varvec{K}}({\varvec{\lambda }})^{\top }{\varvec{p}}= - J'({\varvec{u}})}$, in the case of a linear cost function *J* we obtain

$$\begin{aligned} {\mathcal {J}}({\varvec{\eta }})&= J({{\tilde{{\varvec{u}}}}}) = J({\varvec{u}}) + J'({\varvec{u}})({{\tilde{{\varvec{u}}}}} - {\varvec{u}}) \\&= J({\varvec{u}}) + J'({\varvec{u}})({\varvec{K}}({\varvec{\eta }})^{-1} - {\varvec{K}}({\varvec{\lambda }})^{-1}) {\varvec{f}}\\&= J({\varvec{u}}) - J' ({\varvec{u}})(\eta - {\varvec{\lambda }}_{\ell }){\varvec{K}}({\varvec{\lambda }})^{-1} {\varvec{B}_{\ell }}( {\varvec{I}}_d + (\eta - {\varvec{\lambda }}_{\ell }){\varvec{B}_{\ell }}^\top {\varvec{K}}({\varvec{\lambda }})^{-1} {\varvec{B}_{\ell }})^{-1} {\varvec{B}_{\ell }}^\top {\varvec{K}}({\varvec{\lambda }})^{-1} {\varvec{f}}\\&={\mathcal {J}}({\varvec{\lambda }}) + |T_\ell |(\eta - {\varvec{\lambda }}_{\ell }) (\nabla p_h|_{T_\ell } )^\top ( {\varvec{I}}_d + (\eta - {\varvec{\lambda }}_{\ell }){\varvec{B}_{\ell }}^\top {\varvec{K}}({\varvec{\lambda }})^{-1} {\varvec{B}_{\ell }})^{-1} \nabla u_h|_{T_\ell } \end{aligned}$$

If *J* is not linear the above identity only holds up to a remainder of second order.

From Lemma 3, we get the following result:

#### Proposition 6

Let ${\varvec{\lambda }}, {\varvec{\eta }}\in {\mathbb {R}}^m$. The function $\hat{{\mathcal {J}}}_{\text {SMW}}$ defined by

32 $$\begin{aligned} \hat{{\mathcal {J}}}_{\text {SMW}}({\varvec{\eta }}) :=&{\mathcal {J}}({\varvec{\lambda }}) -\sum _{\ell =1}^m |T_\ell | ({\varvec{\eta }}_\ell - {\varvec{\lambda }}_{\ell }) (\nabla u_h|_{T_\ell })^\top \nonumber \\&( {\varvec{I}}_d + ({\varvec{\eta }}_\ell - {\varvec{\lambda }}_{\ell }){\varvec{B}_{\ell }}^\top {\varvec{K}}({\varvec{\lambda }})^{-1} {\varvec{B}_{\ell }})^{-1} \nabla u_h|_{T_\ell } \end{aligned}$$

is a separable exact model of ${\mathcal {J}}$ at ${\varvec{\lambda }}$.

#### Proof

From Lemma 3 we know that

33 $$\begin{aligned} \hat{{\mathcal {J}}}_{\text {SMW}}({\varvec{\eta }}) = {\mathcal {J}}({\varvec{\lambda }}) + \sum _{\ell =1}^m \left( {\mathcal {J}}({\varvec{\lambda }}+ ({\varvec{\eta }}_\ell - {\varvec{\lambda }}_\ell ) {\varvec{e}}^{(\ell )}) - {\mathcal {J}}({\varvec{\lambda }}) \right) \end{aligned}$$

is a separable exact model of ${\mathcal {J}}$ at ${\varvec{\lambda }}$. Plugging in the result of Proposition 5 yields the assertion. $\square$

Compared to the general separable exact model according to Lemma 3, which can be defined for any function, using the Sherman–Morrison–Woodbury formula we have found a closed form for a separable exact model for our given cost function in (32). Of course, it can be seen that model (32) still involves the inverse of the stiffness matrix for the material distribution given by ${\varvec{\lambda }}$ which one typically does not have access to. Even when the stiffness matrix has been factorized for computing the state ${\varvec{u}}$, the evaluation of (32) involves *m* many forward/backward substitutions which amounts to a total effort in the order of ${\mathcal {O}}(m n^2)$ and is thus prohibitive for many real-world applications. In the subsequent section, we will introduce an approximation of (32) which can be evaluated efficiently.

## An Efficient Separable Model Based on the Sherman–Morrison–Woodbury Formula

Recall the separable exact first order model (32) which reads

34 $$\begin{aligned} \hat{{\mathcal {J}}}_{\text {SMW}}({\varvec{\eta }}) = {\mathcal {J}}({\varvec{\lambda }}) -\sum _{\ell =1}^m |T_\ell | ({\varvec{\eta }}_\ell - {\varvec{\lambda }}_{\ell }) (\nabla u_h|_{T_\ell })^\top \left( {\varvec{I}}_d - ({\varvec{\eta }}_\ell - {\varvec{\lambda }}_{\ell }) {\varvec{\Gamma }}^{(\ell )}\right) ^{-1} \nabla u_h|_{T_\ell } \end{aligned}$$

with the definition ${\varvec{\Gamma }}^{(\ell )}:= - {\varvec{B}_{\ell }}^\top {\varvec{K}}({\varvec{\lambda }})^{-1} {\varvec{B}_{\ell }}\in {\mathbb {R}}^{d\times d}$ for all $\ell \in \{1, \dots , m\}$. Since the evaluation of the model involves the solution of a linear system with the system matrix ${\varvec{K}}({\varvec{\lambda }})$ for every element index $\ell$, we introduce an approximation which can be evaluated more efficiently. For that purpose, we simply approximate the inverse of the stiffness matrix by the inverse of the diagonal approximation of the stiffness matrix to obtain the model

35 $$\begin{aligned} \hat{{\mathcal {J}}}_{\text {SMWdiag}}({\varvec{\eta }}) := {\mathcal {J}}({\varvec{\lambda }}) -\sum _{\ell =1}^m |T_\ell | ({\varvec{\eta }}_\ell - {\varvec{\lambda }}_{\ell }) (\nabla u_h|_{T_\ell })^\top \left( {\varvec{I}}_d - ({\varvec{\eta }}_\ell - {\varvec{\lambda }}_{\ell }) {\varvec{\Gamma }}^{(\ell )}_\text {diag}\right) ^{-1} \nabla u_h|_{T_\ell } \end{aligned}$$

with

36 $$\begin{aligned} {\varvec{\Gamma }}^{(\ell )}_\text {diag} := - {\varvec{B}_{\ell }}^\top (\text{ diag }{\varvec{K}}({\varvec{\lambda }}))^{-1} {\varvec{B}_{\ell }}\in {\mathbb {R}}^{d\times d}. \end{aligned}$$

This model is a separable first order model, but it is no longer exact. Note that this idea was already proposed in [19] in the context of a discrete dipole approximation method in an application from optics and is transferred to a finite element setting here.

In the following, we investigate model (35) in the one-dimensional case, where connections to the mathematical concepts of topological and shape derivatives can be established. In spatial dimension $d=1$, we can compute the matrix ${\varvec{\Gamma }}^{(\ell )}_\text {diag}$ explicitly.

### Lemma 7

Let $d=1$ and let a uniform mesh $\{T_1, \dots , T_m\}$ of the computational domain be given. Assume that element $T_\ell$ is occupied by material $\lambda ^{\textrm{out}}$ and also its two neighbors $T_{\ell -1}$, $T_{\ell +1}$ are occupied by the same material, i.e., ${\varvec{\lambda }}_{\ell -1} = {\varvec{\lambda }}_{\ell } = {\varvec{\lambda }}_{\ell +1} = \lambda ^{\textrm{out}}$. Then it holds

37 $$\begin{aligned} {\varvec{\Gamma }}^{(\ell )}_\text {diag} = -\frac{1}{\lambda ^{\textrm{out}}} \end{aligned}$$

and, for $${\varvec{\eta }}= {\varvec{\lambda }}+ (\lambda ^{\textrm{in}}- \lambda ^{\textrm{out}}){\varvec{e}}^{(\ell )}$$,

38 $$\begin{aligned} \hat{{\mathcal {J}}}_{\text {SMWdiag}}({\varvec{\eta }}) = {\mathcal {J}}({\varvec{\lambda }}) - |T_\ell |\frac{\lambda ^{\textrm{out}}}{\lambda ^{\textrm{in}}} (\lambda ^{\textrm{in}}- \lambda ^{\textrm{out}}) (u_h'|_{T_\ell })^2. \end{aligned}$$

### Proof

Using the definition of ${\varvec{B}_{\ell }}$ from Sect. 2.1.2, we have

$$\begin{aligned} {\varvec{B}_{\ell }}^\top (\text{ diag } {\varvec{K}}({\varvec{\lambda }}))^{-1} {\varvec{B}_{\ell }}= h^{-1} \left( \begin{array}{c} -1 \\ 1 \end{array} \right) ^\top ({{\tilde{\varvec{B}}}_\ell })^\top (\text{ diag } {\varvec{K}}({\varvec{\lambda }}))^{-1} {{\tilde{\varvec{B}}}_\ell }\left( \begin{array}{c} -1 \\ 1 \end{array} \right) \end{aligned}$$

where *h* denotes the uniform mesh size. Using that $(\text{ diag } {\varvec{K}}({\varvec{\lambda }}) )^{-1}_{ii} =\frac{1}{a(\varphi _i, \varphi _i)}$ with $a(\varphi _i, \varphi _i ) = \int \nolimits _{\textsf{D}}\lambda (x) |\nabla \varphi _i|^2 \; \text{ d }x$ and

39 $$\begin{aligned} ({{\tilde{\varvec{B}}}_\ell })^\top (\text{ diag } {\varvec{K}}({\varvec{\lambda }}))^{-1} {{\tilde{\varvec{B}}}_\ell }= \left( \begin{array}{cc} \frac{1}{a(\varphi _{\ell ,1}, \varphi _{\ell ,1})} &  0\\ 0&  \frac{1}{a(\varphi _{\ell ,2}, \varphi _{\ell ,2})} \end{array}\right) \end{aligned}$$

where $\varphi _{\ell ,1}$ and $\varphi _{\ell ,2}$ are the basis functions corresponding to the left and right node of element $T_\ell$, respectively, we get

40 $$\begin{aligned} {\varvec{\Gamma }}^{(\ell )}_\text {diag} = - {\varvec{B}_{\ell }}^\top (\text{ diag } {\varvec{K}}({\varvec{\lambda }}))^{-1} {\varvec{B}_{\ell }}= -h^{-1} \left( \frac{1}{a(\varphi _{\ell ,1}, \varphi _{\ell ,1})} + \frac{1}{a(\varphi _{\ell ,2}, \varphi _{\ell ,2})} \right) . \end{aligned}$$

The first result follows by noting that $a(\varphi _{\ell ,1}, \varphi _{\ell ,1}) = a(\varphi _{\ell ,2}, \varphi _{\ell ,2}) = \frac{2 \lambda ^{\textrm{out}}}{h}$. The second identity follows by plugging in and noting that ${\varvec{\eta }}_\ell = \lambda ^{\textrm{in}}$, ${\varvec{\lambda }}_\ell = \lambda ^{\textrm{out}}$. $\square$

### Remark 7

(Relation to topological derivative in 1D) As pointed out in Sect. 2.2, the topological derivative of our optimization problem (5) at a point $z \in {\textsf{D}}{\setminus } {\overline{\Omega }}$ (i.e., where $\lambda _\Omega (z) = \lambda ^{\textrm{out}}$) with respect to $\omega = (-1,1)$ the one-dimensional unit ball reads

41 $$\begin{aligned} d {\mathcal {J}}[\lambda ^{\textrm{out}}, \lambda ^{\textrm{in}}](\Omega )(z, \omega ) = -\frac{\lambda ^{\textrm{out}}}{\lambda ^{\textrm{in}}}(\lambda ^{\textrm{in}}- \lambda ^{\textrm{out}}) (u'(z))^2, \end{aligned}$$

where we used that $p = -u$ for our particular problem at hand. This means that, in one space dimension, the model $\hat{{\mathcal {J}}}_{\text {SMWdiag}}$ introduced in (35) actually coincides with the finite element discretization of the model that is naturally defined by the definition of the topological derivative

42 $$\begin{aligned} {\mathcal {J}}(\Omega \cup \omega _\varepsilon ) \approx {\mathcal {J}}(\Omega ) + |\omega _\varepsilon | d{\mathcal {J}}(\Omega )(z, \omega ), \end{aligned}$$

see also (15).

We mention that this direct correspondence of the discrete model $\hat{{\mathcal {J}}}_{\text {SMWdiag}}$ and the closed-form formula of the topological derivative only works since the elements $T_\ell$ in a 1D mesh are scaled versions of the 1D unit ball $\omega = B_1(0) = (-1,1)$. This is no longer the case in two or three dimensions, where elements are polygonal or polyhedral.

We remark that we also observed numerically that the finite element discretization of (42) and the model (35) coincide in elements $T_\ell$ in homogeneous regions (i.e., where ${\varvec{\lambda }}_{\ell -1}={\varvec{\lambda }}_{\ell }={\varvec{\lambda }}_{\ell +1}$). In elements $T_\ell$ that are adjacent to the material interface $\partial \Omega$, however, Lemma 7 and thus formula (38) are no longer valid. We observed that the topological derivative model (42), however, still yielded very good results. This can be explained by the following discussion on the relation between the topological and shape derivative in 1D.

### Remark 8

(Relation to shape derivative in 1D) The shape derivative for moving the interface $\Gamma := \overline{\Omega }\cap \overline{ ({\textsf{D}}{\setminus } \Omega )}$ in the direction given by a vector field $V \in C^1( {\mathbb {R}}^d, {\mathbb {R}}^d)$ can, by the structure theorem of Hadamard-Zolesio [1], always (under suitable smoothness assumptions) be written in the form

$$\begin{aligned} d {\mathcal {J}}(\Omega ; V) = \int \nolimits _\Gamma L (V \cdot n) \; ds_x, \end{aligned}$$

for some scalar function *L*. Here $\Omega \subset {\textsf{D}}$ is the domain where $\lambda = \lambda ^{\textrm{in}}$ and ${\textsf{D}}{\setminus } \overline{\Omega }$ is where $\lambda = \lambda ^{\textrm{out}}$, and *n* denotes the outer unit normal vector to $\Omega$. For our problem (5), *L* is given by the formula

$$\begin{aligned} L = (\lambda ^{\textrm{in}}- \lambda ^{\textrm{out}}) (\nabla u\cdot \tau )(\nabla p\cdot \tau ) -\left( \frac{1}{\lambda ^{\textrm{in}}} - \frac{1}{\lambda ^{\textrm{out}}} \right) (\lambda _\Omega \nabla u \cdot n) (\lambda _\Omega \nabla p \cdot n) \end{aligned}$$

with the tangential vector $\tau$ (see, e.g., [29]). In the case where $d=1$ the tangential derivative of *u* and thus the first term of *L* vanishes. Using that *n* is a scalar with $n^2=1$ and again that $p = -u$, we get

$$\begin{aligned} L = \left( \frac{1}{\lambda ^{\textrm{in}}} - \frac{1}{\lambda ^{\textrm{out}}} \right) (\lambda ^{\textrm{out}}u_{\text {out}}'|_\Gamma )^2 = -\frac{\lambda ^{\textrm{out}}}{\lambda ^{\textrm{in}}}(\lambda ^{\textrm{in}}- \lambda ^{\textrm{out}}) (u_{\text {out}}'|_\Gamma )^2, \end{aligned}$$

where $u_{\text {out}}'|_\Gamma$ denotes the limit at $\Gamma$ of the discontinuous quantity $u'$ when coming from ${\textsf{D}}{\setminus } {\overline{\Omega }}$.

Note that this formula for the 1D shape derivative resembles the topological derivative formula (41), which explains why (42) is a very good model in 1D.

## A Separable Model Based on the Topological Derivative

In this section, we propose a separable model that is based on the notion of the topological derivative. We fix the space dimension $d=2$. The topological derivative of a shape function ${\mathcal {J}} = {\mathcal {J}}(\Omega )$ with respect to a perturbation of shape $\omega$ around a spatial point *z* was introduced in Sect. 2.2. We emphasize that, while closed-form formulas for the topological derivative only exist in the case of circular or elliptic inclusion shapes [24, 30, 31], a numerical approximation of the weak polarization matrix (20) and thus of the topological derivative formulas (18) and (19) is possible for arbitrary inclusion shapes $\omega$ with $0 \in \omega$, see also [20]. We will follow this idea for the case of triangular inclusion shapes.

**Fig. 2:**
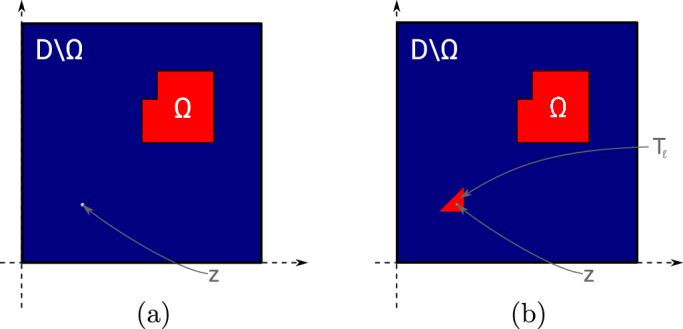
**a** Unperturbed configuration. **b** Perturbed configuration where the domain is perturbed in triangle $T_\ell$ whose centroid is the point *z*

Let $\Omega \subset {\textsf{D}}$ be given and consider an element $T_\ell \in {\mathcal {T}}$ of type 1 (cf. Figure 1) with $T_\ell \subset {\textsf{D}}\setminus {\overline{\Omega }}$ with vertices ${\varvec{x}}_{\ell ,1}, {\varvec{x}}_{\ell ,2}, {\varvec{x}}_{\ell ,3}$ (in counter-clockwise enumeration) and centroid ${\varvec{z}}_\ell := ({\varvec{x}}_{\ell ,1}+{\varvec{x}}_{\ell ,2}+{\varvec{x}}_{\ell ,3})/3$, see Fig. 2. Let ${{\hat{T}}}^{(1)}$ denote the reference triangle defined by its three vertices $\frac{1}{3}(-2,-1)^\top$, $\frac{1}{3}(1,-1)^\top$, $\frac{1}{3}(1,2)^\top$ and ${{\hat{T}}}^{(2)}$ the reference triangle with vertices $\frac{1}{3}(2,1)^\top$, $\frac{1}{3}(-1,-2)^\top$, $\frac{1}{3}(-1,1)^\top$. For the rest of this section we restrict ourselves to elements of type 1. We set ${{\hat{T}}}:= {{\hat{T}}}^{(1)}$ and define $\Phi _{h,\ell }: {{\hat{T}}} \rightarrow T_\ell , {\varvec{x}}\mapsto {\varvec{z}}_\ell + h {\varvec{x}}$ as the affine transformation satisfying $\Phi _{h,\ell }({{\hat{T}}}) = T_\ell$. We remark that the procedure is completely analogous for elements of type 2 using reference triangle ${{\hat{T}}}^{(2)}$.

### Derivation of Topological Derivatives for Triangular Inclusion Shapes

Given a domain $\Omega$, recall the notation $\lambda _\Omega (x) = \chi _\Omega (x) \lambda ^{\textrm{in}}+ \chi _{{\textsf{D}}{\setminus } {\overline{\Omega }}}(x) \lambda ^{\textrm{out}}$. For the fixed triangular domain perturbation $T_\ell$, let the perturbed solution $u^{(\ell )} \in V_g$ be defined as the unique solution satisfying

43 $$\begin{aligned} \int \nolimits _{\textsf{D}}\lambda _{\Omega \cup T_\ell }(x) \nabla u^{(\ell )} \cdot \nabla v \; \text{ d }x = \int \nolimits _{\textsf{D}}f v \; \text{ d }x + \int \nolimits _{\Gamma _N} g_N v \; \text{ d }s_x \end{aligned}$$

for all $v \in V_0$. We rewrite the difference of the perturbed and unperturbed cost function by adding the equations (43) and (5b) defining $u^{(\ell )}$ and *u*, respectively. Using the adjoint state *p* defined by (6) as test function, we obtain

$$\begin{aligned} {\mathcal {J}}(\Omega \cup T_\ell ) -&{\mathcal {J}}(\Omega ) = J(u^{(\ell )}) - J(u) \\ =&J(u^{(\ell )}) + \int \nolimits _{\textsf{D}}\lambda _{\Omega \cup T_\ell }(x) \nabla u^{(\ell )} \cdot \nabla p \; \text{ d }x - \int \nolimits _{\textsf{D}}f p \; \text{ d }x - \int \nolimits _{\Gamma _N} g_N p \; \text{ d }s_x \\&- J(u) - \int \nolimits _{\textsf{D}}\lambda _{\Omega }(x) \nabla u \cdot \nabla p \; \text{ d }x + \int \nolimits _{\textsf{D}}f p \; \text{ d }x + \int \nolimits _{\Gamma _N} g_N p \; \text{ d }s_x \\ =&\int \nolimits _{\textsf{D}}f (u^{(\ell )} - u) \; \text{ d }x + \int \nolimits _{\Gamma _N} g_N (u^{(\ell )} - u) \; \text{ d }s_x \\&+ \int \nolimits _{\textsf{D}}\lambda _{\Omega }(x) \nabla (u^{(\ell )}-u) \cdot \nabla p \; \text{ d }x + (\lambda ^{\textrm{in}}- \lambda ^{\textrm{out}}) \int \nolimits _{T_\ell } \nabla u^{(\ell )} \cdot \nabla p \; \text{ d }x \\ =&(\lambda ^{\textrm{in}}- \lambda ^{\textrm{out}}) \int \nolimits _{T_\ell } \nabla (u^{(\ell )}-u) \cdot \nabla p \; \text{ d }x + (\lambda ^{\textrm{in}}- \lambda ^{\textrm{out}}) \int \nolimits _{T_\ell } \nabla u \cdot \nabla p \; \text{ d }x \end{aligned}$$

where we used the adjoint equation (6) in the last step. Making a change of variables $x \mapsto \Phi _{h,\ell }(x)$ and defining $K_{h,\ell , {{\hat{T}}}}[\lambda ^{\textrm{out}}, \lambda ^{\textrm{in}}]:= \frac{1}{h} (u^{(\ell )} - u)\circ \Phi _{h,\ell }$, we get

44 $$\begin{aligned} \begin{aligned} {\mathcal {J}}(\Omega \cup T_\ell ) =&{\mathcal {J}}(\Omega ) + h^2 (\lambda ^{\textrm{in}}- \lambda ^{\textrm{out}}) \int \nolimits _{{{\hat{T}}}} \nabla K_{h,\ell , {{\hat{T}}}}[\lambda ^{\textrm{out}}, \lambda ^{\textrm{in}}] \cdot (\nabla p)\circ \Phi _{h,\ell } \; \text{ d }x \\&+ h^2(\lambda ^{\textrm{in}}- \lambda ^{\textrm{out}}) \int \nolimits _{{{\hat{T}}}} (\nabla u)\circ \Phi _{h,\ell } \cdot (\nabla p)\circ \Phi _{h,\ell } \; \text{ d }x, \end{aligned} \end{aligned}$$

where we used that, according to the chain rule, $(\nabla v)\circ \Phi _{h,\ell } = \frac{1}{h} \nabla (v\circ \Phi _{h,\ell })$ and $\text {det}(\partial \Phi _{h,\ell }) = h^2$.

**Fig. 3:**
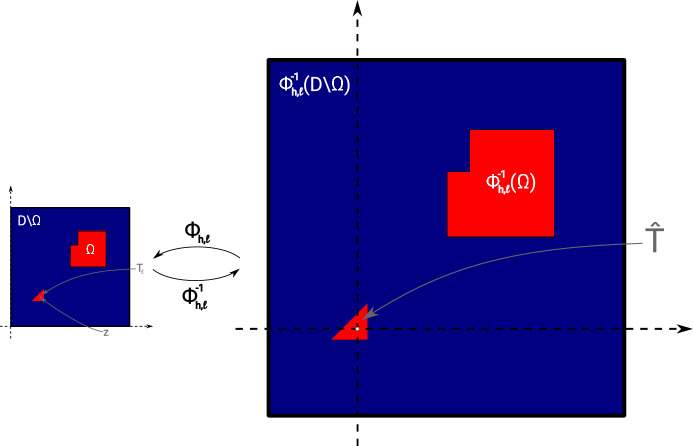
Rescaled perturbed domain

Subtracting the unperturbed state equation (5b) from the perturbed equation (43), we see that $u^{(\ell )} - u \in V_0$ satisfies

$$\begin{aligned} \int \nolimits _{\textsf{D}}\lambda _{\Omega \cup T_\ell } \nabla (u^{(\ell )} - u ) \cdot \nabla v \; \text{ d }x = -(\lambda ^{\textrm{in}}- \lambda ^{\textrm{out}}) \int \nolimits _{T_\ell } \nabla u \cdot \nabla v \; \text{ d }x \end{aligned}$$

for all $v \in V_0$. Making the same change of variables, this amounts to

45 $$\begin{aligned}&\int \nolimits _{\Phi _{h,\ell }^{-1}({\textsf{D}})}\lambda _{{{\hat{T}}} \cup \Phi _{h,\ell }^{-1}(\Omega )} \nabla K_{h,\ell , {{\hat{T}}}}[\lambda ^{\textrm{out}}, \lambda ^{\textrm{in}}] \cdot \nabla \psi \; \text{ d }x \nonumber \\&\quad = -(\lambda ^{\textrm{in}}- \lambda ^{\textrm{out}}) \int \nolimits _{{{\hat{T}}}} (\nabla u)\circ \Phi _{h,\ell } \cdot \nabla \psi \; \text{ d }x \end{aligned}$$

for all $\psi \in H^1_0(\Phi _{h,\ell }^{-1}({\textsf{D}}))$. The domain and material distribution of problem (45) is depicted in Fig. 3.

**Fig. 4:**
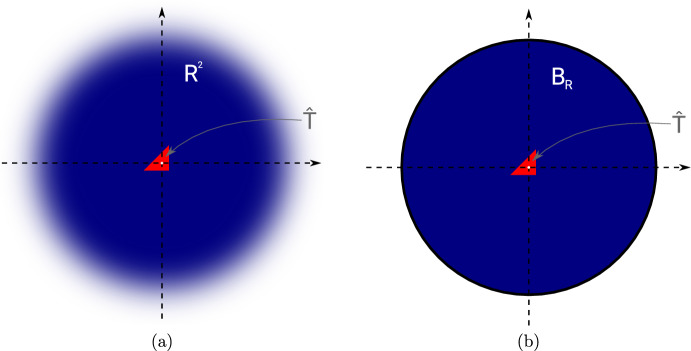
**a** Rescaled perturbed domain after limit $h \rightarrow 0$. **b** Truncation of (**a**) at radius *R*

Obviously, if $K_{h,\ell , {{\hat{T}}}}[\lambda ^{\textrm{out}}, \lambda ^{\textrm{in}}]$ was known exactly then (44) would give rise to an exact model for ${\mathcal {J}}(\Omega \cup T_\ell )$. However, of course, this would require the solution of a linear problem for every element $\ell$ and is therefore computationally not tractable. Instead, we now aim at obtaining an approximation of the quantity $K_{h,\ell , {{\hat{T}}}}[\lambda ^{\textrm{out}}, \lambda ^{\textrm{in}}]$ that is independent of the particular element index $\ell$. As it is often used in the derivation of topological derivatives, we consider the limit of problem (45) as $h \rightarrow 0$. This leads to the problem to find $K_{{{\hat{T}}}}[\lambda ^{\textrm{out}}, \lambda ^{\textrm{in}}; \nabla u(z_\ell )] \in X$ satisfying [22]

46 $$\begin{aligned} \int \nolimits _{{\mathbb {R}}^2}\lambda _{{{\hat{T}}} } \nabla K_{{{\hat{T}}}}[\lambda ^{\textrm{out}}, \lambda ^{\textrm{in}};\nabla u(z_\ell )] \cdot \nabla \psi \; \text{ d }x = -(\lambda ^{\textrm{in}}- \lambda ^{\textrm{out}}) \int \nolimits _{{{\hat{T}}}} \nabla u(z_\ell ) \cdot \nabla \psi \; \text{ d }x \end{aligned}$$

for all test functions $\psi \in X$ where *X* is a Beppo-Levi space, see Sect. 2.2. For an illustration of the corresponding material distribution, see Fig. 4a. Note that, if we had access to the exact solution $K_{{{\hat{T}}}}[\lambda ^{\textrm{out}}, \lambda ^{\textrm{in}};\nabla u(z_\ell )]$ of (46), the topological derivative at the centroid $z_\ell$ of triangle $T_\ell \in {\textsf{D}}{\setminus } \overline{\Omega }$ with respect to ${{\hat{T}}}$-shaped inclusion shapes would follow from (44) as

47 $$\begin{aligned}&d {\mathcal {J}}[\lambda ^{\textrm{out}}, \lambda ^{\textrm{in}}](\Omega )(z_\ell , {{\hat{T}}}) \nonumber \\&\quad = \underset{h \rightarrow 0}{\text{ lim }} \frac{{\mathcal {J}}(\Omega \cup T_\ell ) - {\mathcal {J}}(\Omega ) }{|T_\ell |} \nonumber \\&\quad = (\lambda ^{\textrm{in}}- \lambda ^{\textrm{out}}) \frac{1}{|{{\hat{T}}}|} \int \nolimits _{{{\hat{T}}}} (\nabla u(z_\ell ) + \nabla K_{{{\hat{T}}}}[\lambda ^{\textrm{out}}, \lambda ^{\textrm{in}};\nabla u(z_\ell )](x)) \cdot \nabla p(z_\ell ) \; \text{ d }x \end{aligned}$$

which coincides with the statement of Proposition 2. Here we used $|T_\ell | = h^2|{{\hat{T}}}|$.

### Our Proposed Topological Derivative Model

Unlike in the case of circular or elliptic inclusions, no closed-form solution to the exterior problem (46) for triangular inclusion shapes $\omega = {{\hat{T}}}$ is known in the literature. Thus, formula (47) cannot be evaluated exactly. However, as it was shown in [20], it is feasible to numerically approximate the exterior problem (46) by truncating the domain at a moderately large radius *R* (e.g., $R=30$) and using a finite element discretization with homogeneous Dirichlet boundary conditions on the boundary of the truncated domain (see Fig. 4b). We remark that this truncation is justified, since it is known that the solution to (46) exhibits a certain decay as $|x| \rightarrow \infty$ [20].

#### Topological Derivative Model in Homogeneous Regions

We restrict ourselves to elements $T_\ell$ in the interior of ${\textsf{D}}{\setminus } {\overline{\Omega }}$ such that also all neighboring elements of $T_\ell$ (i.e., elements that share at least one vertex with $T_\ell$) are in ${\textsf{D}}\setminus {\overline{\Omega }}$. Of course, all results and statements follow analogously for elements $T_\ell$ in the interior of $\Omega$. For this setting, we propose the model that is based on the following procedure:

1. Compute a finite element approximation of (46) using a finite element discretization of a truncated domain. More precisely, given a truncation radius *R* and a mesh $\{\tau _1, \dots , \tau _M\}$ of the truncated domain $B_R(0)$ that resolves the inclusion ${{\hat{T}}}$, we aim to find $K_{{{\hat{T}}},h}[\lambda ^{\textrm{out}}, \lambda ^{\textrm{in}};{\varvec{e}}^{(k)}] \in V_{h}^R:= \{v \in C^0(B_R(0)): v|_{\partial B_R(0)}=0, v|_{\tau _i} \in P^1, i=1,\dots , M\}$ such that
  48 $$\begin{aligned} \int \nolimits _{B_R(0)}\lambda _{{{\hat{T}}} } \nabla K_{{{\hat{T}}},h}[\lambda ^{\textrm{out}}, \lambda ^{\textrm{in}};{\varvec{e}}^{(k)}] \cdot \nabla \psi _h \; \text{ d }x = -(\lambda ^{\textrm{in}}- \lambda ^{\textrm{out}}) \int \nolimits _{{{\hat{T}}}} {\varvec{e}}^{(k)} \cdot \nabla \psi _h \; \text{ d }x \end{aligned}$$
  for all $\psi _h \in V_{h}^R$ for $k =1$ and $k=2$. Here, recall that $\lambda _{{{\hat{T}}}}(x) = \chi _{{{\hat{T}}}}(x) \lambda ^{\textrm{in}}+ \chi _{{\mathbb {R}}^2 {\setminus } {{\hat{T}}}}(x) \lambda ^{\textrm{out}}$.
2. Compute the approximate weak polarization matrix
  49 $$\begin{aligned}&{\mathcal {P}}_{{{\hat{T}}}, h}[\lambda ^{\textrm{out}}, \lambda ^{\textrm{in}}] \nonumber \\&\quad = \left[ \frac{1}{|{{\hat{T}}}|} \int \nolimits _{{{\hat{T}}}}\nabla K_{{{\hat{T}}},h}[\lambda ^{\textrm{out}}, \lambda ^{\textrm{in}};{\varvec{e}}^{(1)}]\text{ d }x \quad \frac{1}{|{{\hat{T}}}|} \int \nolimits _{{{\hat{T}}}} \nabla K_{{{\hat{T}}},h}[\lambda ^{\textrm{out}}, \lambda ^{\textrm{in}};{\varvec{e}}^{(2)}] \text{ d }x \right] \in \mathbb R^{2\times 2}. \end{aligned}$$
3. Evaluate $$d {\mathcal {J}}_h[\lambda ^{\textrm{out}}, \lambda ^{\textrm{in}}](z_\ell , {{\hat{T}}}) = -(\lambda ^{\textrm{in}}- \lambda ^{\textrm{out}}) (\nabla u_h|_{T_\ell })^\top \left( {\varvec{I}}_2 + {\mathcal {P}}_{{{\hat{T}}}, h}[\lambda ^{\textrm{out}}, \lambda ^{\textrm{in}}] \right) \nabla u_h|_{T_\ell }$$.

With this, for a given material distribution ${\varvec{\lambda }}\in {\mathbb {R}}^m$ and ${\varvec{\eta }}= {\varvec{\lambda }}+ (\eta - {\varvec{\lambda }}_\ell ) {\varvec{e}}^{(\ell )}$ where $T_\ell$ is in the interior of ${\textsf{D}}\setminus {\overline{\Omega }}$, we obtain the approximation

$$\begin{aligned} {\mathcal {J}}({\varvec{\eta }}) \approx&{\mathcal {J}}({\varvec{\lambda }}) + |T_\ell | d {\mathcal {J}}_h[{\varvec{\lambda }}_\ell ,\eta ](z_\ell , {{\hat{T}}}) \\ =&{\mathcal {J}}({\varvec{\lambda }}) - |T_\ell |(\eta - {\varvec{\lambda }}_\ell ) (\nabla u_h|_{T_\ell })^\top \left( {\varvec{I}}_2 + {\mathcal {P}}_{{{\hat{T}}}, h}[{\varvec{\lambda }}_\ell , \eta ] \right) \nabla u_h|_{T_\ell }. \end{aligned}$$

##### Remark 9

**Fig. 5:**
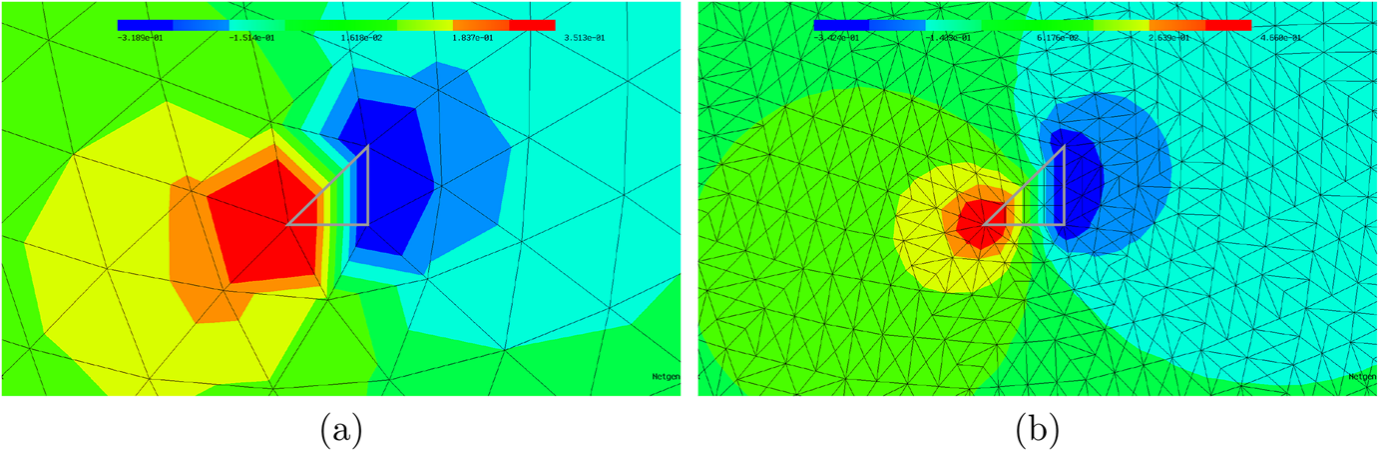
Comparison of numerical solution $K_{{{\hat{T}}}, h}[1, 1000;{\varvec{e}}^{(1)}]$ to (48) on a mesh where ${{\hat{T}}}$ is resolved by exactly one element (**a**) and on a twice uniformly refined mesh. The mesh in (a) should be used for solving (48)

Concerning the numerical solution of (48), we make one important remark. It is essential that the mesh $\{\tau _1, \dots , \tau _M\}$ is chosen in such a way that the triangle ${{\hat{T}}}$ is discretized by exactly one element $\tau _j$ and that, thus, the solution is linear inside the whole of ${{\hat{T}}}$. While a finer discretization of ${{\hat{T}}}$ would yield a better approximation to the true solution of limit problem (46), the term $K_{{{\hat{T}}},h}[\lambda ^{\textrm{out}}, \lambda ^{\textrm{in}}; {\varvec{e}}^{(k)}]$ should actually make up for the error $u_h^{(\ell )} - u_h$ inside element $T_\ell$ which is a linear function inside $T_\ell$ due to the chosen discretization. Figure 5 shows the solution to (48) when ${{\hat{T}}}$ is resolved by exactly one element and when the whole mesh is twice uniformly refined.

#### Topological Derivative Model in Inhomogeneous Regions

Even if one is interested in binary designs without intermediate materials, in the course of a density-based topology or material optimization procedure, one will of course always encounter regions of intermediate materials. For elements $T_\ell$ in these regions, the assumptions taken at the beginning of Sect. 4.2.1 are not satisfied and the corresponding proposed model will not be very accurate in these regions. In order to improve the quality of the approximation also in these regions, we recall the idea behind the topological derivative model: The quantity $K_{{{\hat{T}}}}$ should approximate the local variation of the (discretized) state with respect to a material perturbation in some element $T_\ell$, i.e., it should approximate $u_h^{(\ell )}-u_h$. In other words, problem (46) can be interpreted as considering $u_h^{(\ell )}-u_h$ and zooming in around the fixed triangle $T_\ell$ and neglecting everything that is beyond a certain (small) distance from that triangle, see also the illustrations in Figs. 3 and 4.

**Fig. 6:**
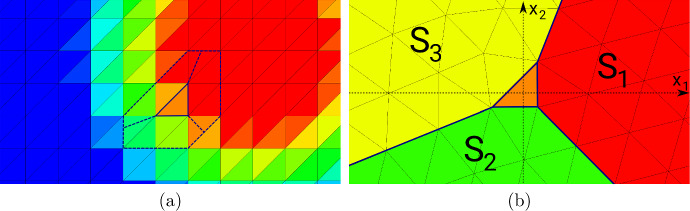
**a** Element inside inhomogeneous material distribution in computational domain ${\textsf{D}}$. Neighborhood for averaging into sector values is marked. **b** Averaged material distribution in three sectors of truncated unbounded domain $B_R(0)$. The average values per sector are obtained by a weighted Hölder mean of the material values in the neighboring elements

We follow this idea also in the case of inhomogeneous material around a fixed triangle, i.e., we want to approximate the local material distribution in a truncated rescaled domain $B_R(0)$ similar to Fig. 4b. For obtaining an approximation of the inhomogeneous material distribution within the computational domain ${\textsf{D}}$, we divide the domain $B_R(0)$ into three sectors. The sectors are separated by three lines which are chosen such as to halve the three interior angles of the triangle ${{\hat{T}}}$. Thus, we end up with a domain $B_R(0)$ similar to the one depicted in Fig. 4b which is occupied by four different materials (one inside the triangle ${{\hat{T}}}$ and one in each of the three sectors), see Fig. 6b. For the computation of average values within one of the sectors, we take a weighted Hölder average of the values in the neighboring elements with parameter $\alpha$, i.e., the averaged value in Sector *j*, $j=1,2,3$, is chosen as

50 $$\begin{aligned} \lambda ^{S_j}_{T_\ell } = \left( \sum _{T \in {\mathcal {N}}(T_\ell )} w_{S_j, T} ({\varvec{\lambda }}_T)^\alpha \right) ^{\frac{1}{\alpha }}. \end{aligned}$$

Here, ${\mathcal {N}}(T_\ell )$ denotes the set of triangles that have at least one common vertex with triangle $T_\ell$, see Fig. 6a, and $w_{S_j, T} = |T \cap S_j|/|T| \in [0,1]$ is the volume fraction of triangle *T* in Sector $S_j$. Moreover, ${\varvec{\lambda }}_T$ denotes the entry of the vector ${\varvec{\lambda }}$ corresponding to the triangle *T*. In our experiments, we chose the Hölder parameter as $\alpha =-0.5$. This choice will be motivated later in Remark 13 of Sect. 7.

Our proposed model in the case of inhomogeneous material around an element $T_\ell$ with material coefficient ${\varvec{\lambda }}_\ell$ and averaged sector values $\lambda ^{S_1}_{T_\ell }, \lambda ^{S_2}_{T_\ell },\lambda ^{S_3}_{T_\ell }$ according to (50) follows the same three steps outlined for the homogeneous setting in Sect. 4.2.1: We numerically compute the corresponding correctors $K_{{{\hat{T}}}, h}[(\lambda ^{\textrm{out}}, \lambda ^{S_1}_{T_\ell }, \lambda ^{S_2}_{T_\ell },\lambda ^{S_3}_{T_\ell }), \lambda ^{\textrm{in}}; {\varvec{e}}^{(k)}]$ for $k=1,2$ as the finite element solutions to (48) where $\lambda _{{{\hat{T}}}}$ is replaced by the new three-sector material distribution

51 $$\begin{aligned} \lambda _{{{\hat{T}}}}(x) = \chi _{{{\hat{T}}}}(x) \lambda ^{\textrm{in}}+ \sum _{j=1}^3\chi _{S_j}(x) \lambda ^{S_j}_{T_\ell }. \end{aligned}$$

Subsequently, the corresponding weak polarization matrix ${\mathcal {P}}_{{{\hat{T}}}, h}[(\lambda ^{\textrm{out}}, \lambda ^{S_1}_{T_\ell }, \lambda ^{S_2}_{T_\ell },\lambda ^{S_3}_{T_\ell }), \lambda ^{\textrm{in}}]$ and the quantity $d{\mathcal {J}}_h[(\lambda ^{\textrm{out}}, \lambda ^{S_1}_{T_\ell }, \lambda ^{S_2}_{T_\ell },\lambda ^{S_3}_{T_\ell }), \lambda ^{\textrm{in}}](z_\ell , {{\hat{T}}})$ can be computed according to steps 2 and 3 of Sect. 4.2.1.

Summarizing, we define the model

52 $$\begin{aligned} \hat{{\mathcal {J}}}_{\text {TDnum}}({\varvec{\eta }}) :=&{\mathcal {J}}({\varvec{\lambda }}) - \sum _{\ell = 1}^m |T_\ell |({\varvec{\eta }}_\ell - {\varvec{\lambda }}_\ell ) (\nabla u_h|_{T_\ell })^\top \nonumber \\&\left( {\varvec{I}}_2 + {\mathcal {P}}_{{{\hat{T}}}, h}[({\varvec{\lambda }}_\ell , \lambda ^{S_1}_{T_\ell }, \lambda ^{S_2}_{T_\ell },\lambda ^{S_3}_{T_\ell }), {\varvec{\eta }}_\ell ] \right) \nabla u_h|_{T_\ell }. \end{aligned}$$

Note that the homogeneous setting of Sect. 4.2.1 is actually also covered by the more general inhomogeneous setting treated here.

We next make an important remark concerning the efficient evaluation of the model $\hat{{\mathcal {J}}}_{\text {TDnum}}$.

##### Remark 10

In the general inhomogeneous setting, the procedure described in this section states that, in order to evaluate model (52), a problem of type (48) has to be solved for each element $T_\ell$. Of course, this is computationally expensive and therefore not recommended by the authors. Instead, the approach followed here is to divide the numerical computations into an offline and an online stage. In the offline stage, which has to be performed only once for the particular type of PDE operator, we compute the quantities

$$\begin{aligned} K_{{{\hat{T}}}, h}[(\lambda ^{\textrm{out}}, \lambda ^{S_1}, \lambda ^{S_2},\lambda ^{S_3}), \lambda ^{\textrm{in}}; {\varvec{e}}^{(k)}] \end{aligned}$$

for $k=1,2$ and for a large number of combinations of relevant values $(\lambda ^{\textrm{out}}, \lambda ^{S_1}_{T_\ell }, \lambda ^{S_2}_{T_\ell },\lambda ^{S_3}_{T_\ell }, \lambda ^{\textrm{in}})$ and store the corresponding weak polarization matrices. This, initially, results in a five-dimensional array of $2\times 2$ matrices. Moreover, these precomputations should be done for each type of reference triangle, i.e., in our case for ${{\hat{T}}} = {{\hat{T}}}^{(1)}$ and ${{\hat{T}}} = {{\hat{T}}}^{(2)}$, see Fig. 1.

In the online stage, for each element $T_\ell$ the corresponding average sector values are computed according to (50) and the weak polarization matrix ${\mathcal {P}}_{{{\hat{T}}}, h}[({\varvec{\lambda }}_\ell , \lambda ^{S_1}_{T_\ell }, \lambda ^{S_2}_{T_\ell },\lambda ^{S_3}_{T_\ell }), {\varvec{\eta }}_\ell ]$ is approximately obtained by piecewise linear interpolation of the precomputed values.

We finally remark that the precomputation can be reduced from five to four dimensions by exploiting that problem (48) with $\lambda _{{{\hat{T}}}}$ according to (51) depends on the parameter $\lambda ^{\textrm{out}}$ only via the scaling of the right hand side by $(\lambda ^{\textrm{in}}- \lambda ^{\textrm{out}})$.

## An Improved Separable Model Based on the Sherman–Morrison–Woodbury Formula

In this section, we revisit the separable exact model defined in (32) and have a closer look at the matrix

$$\begin{aligned} {\varvec{\Gamma }}^{(\ell )} = -{\varvec{B}_{\ell }}^\top {\varvec{K}}({\varvec{\lambda }})^{-1} {\varvec{B}_{\ell }}\in \mathbb R^{2\times 2}. \end{aligned}$$

Recall that, in order to employ model (32), this matrix would have to be evaluated for each element index $\ell$, which amounts to solving *m* many systems of linear equations and is thus computationally prohibitive. Motivated by the procedure of Sect. 4, our goal here is to find a good approximation of ${\varvec{\Gamma }}^{(\ell )}$ that is independent of the element index $\ell$ and can thus be precomputed in an offline stage.

We begin by making the following observation. We assume the finite element setting introduced in Sect. 2.1.2 with the mesh ${\mathcal {T}}$ and the finite element space $V_h\subset H^1_{\Gamma _D}({\textsf{D}})$ of piecewise linear and globally continuous functions.

### Lemma 8

Let $T_\ell \in {\mathcal {T}}$ and, for $k=1,2$, define $w_{k,h} \in V_h$ the unique numerical solution to the variational problem

53 $$\begin{aligned} \int \nolimits _D \lambda (x) \nabla w_{k,h} \cdot \nabla v_h \; \text{ d }x = - \int \nolimits _{T_\ell } {\varvec{e}}^{(k)} \cdot \nabla v_h \; \text{ d }x \end{aligned}$$

for all $v_h \in V_h$. Then it holds

54 $$\begin{aligned} {\varvec{\Gamma }}^{(\ell )} = [ \nabla w_{1,h}|_{T_\ell }\; \nabla w_{2,h}|_{T_\ell } ] . \end{aligned}$$

### Proof

We use the notation and symbols introduced in Sect. 2.1.2. The discretization of (53) reads

$$\begin{aligned} {\varvec{K}}({\varvec{\lambda }}) {\varvec{w}}^{(k)} = {\varvec{f}}^{(\ell ,k)} \end{aligned}$$

where ${\varvec{K}}({\varvec{\lambda }})$ is the invertible stiffness matrix, ${\varvec{w}}^{(k)}$ denotes the coefficient vector of the finite element function $w_{k,h} = \sum _{i=1}^n {\varvec{w}}^{(k)}_i \varphi _i$, $k\in \{1,2\}$, and ${\varvec{f}}^{(\ell ,k)} \in {\mathbb {R}}^n$ with

$$\begin{aligned} ({\varvec{f}}^{(\ell ,k)})_i =&-\int \nolimits _{T_\ell } {\varvec{e}}^{(k)} \cdot \nabla \varphi _i \; \text{ d }x, \; i=1,\dots n. \end{aligned}$$

Since the global load vector ${\varvec{f}}^{(\ell , k)}$ has contributions only from one element, it holds ${\varvec{f}}^{(\ell , k)} = {{\tilde{\varvec{B}}}_\ell }{\varvec{f}}^{\ell , k}_{\text {loc}}$ with the element load vector

$$\begin{aligned} {\varvec{f}}^{\ell , k}_{\text {loc}} = - |T_\ell | \begin{pmatrix} -1 &  -1 \\ 1 &  0 \\ 0 &  1 \end{pmatrix} {\varvec{J}}_\ell ^{-1} {\varvec{e}}^{(k)} = - \sqrt{|T_\ell |} {\varvec{D}}_\ell {\varvec{e}}^{(k)}. \end{aligned}$$

Thus, it holds

55 $$\begin{aligned} {\varvec{B}_{\ell }}^\top {\varvec{w}}^{(k)} = {\varvec{B}_{\ell }}^\top {\varvec{K}}({\varvec{\lambda }})^{-1} {\varvec{f}}^{(\ell ,k)} = {\varvec{B}_{\ell }}^\top {\varvec{K}}({\varvec{\lambda }})^{-1} {{\tilde{\varvec{B}}}_\ell }{\varvec{f}}^{\ell , k}_{\text {loc}} = - \sqrt{|T_\ell |} {\varvec{B}_{\ell }}^\top {\varvec{K}}({\varvec{\lambda }})^{-1} {\varvec{B}_{\ell }}{\varvec{e}}^{(k)}. \end{aligned}$$

On the other hand, we know from (13) that

56 $$\begin{aligned} {\varvec{B}_{\ell }}^\top {\varvec{w}}^{(k)} = \sqrt{|T_\ell |} \, \nabla w_{k,h}|_{T_\ell }. \end{aligned}$$

Comparing (55) and (56) for $k=1$ and $k=2$ yields the result. $\square$

**Fig. 7:**
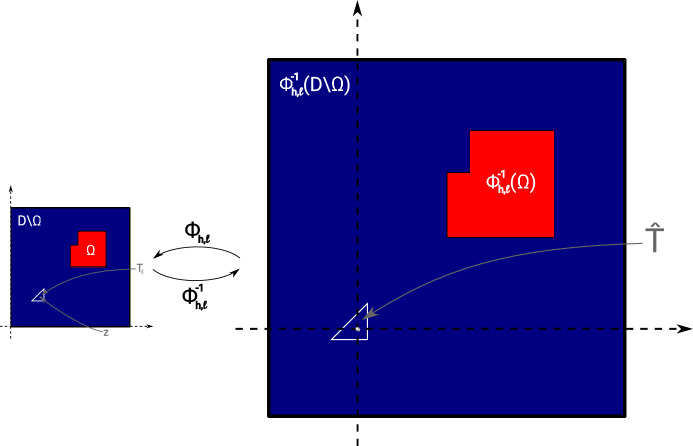
Rescaled unperturbed domain

Lemma 8 gives an interpretation of the matrix ${\varvec{\Gamma }}^{(\ell )}$, which appears in the Sherman–Morrison–Woodbury model (32) and is costly to evaluate, in terms of a boundary value problem. In order to find an approximation of ${\varvec{\Gamma }}^{(\ell )}$ that is independent of the element index $\ell$, we proceed similarly to Sect. 4. In boundary value problem (53), we zoom in around the element $T_\ell$, i.e., we apply the transformation $\Phi _{h,\ell }^{-1}$ that transforms $T_\ell$ to the reference element ${{\hat{T}}}$, see Fig. 7 for an illustration in a homogeneous setting. Note that, as opposed to the procedure in Sect. 4, here an unperturbed material distribution is transformed.

**Fig. 8:**
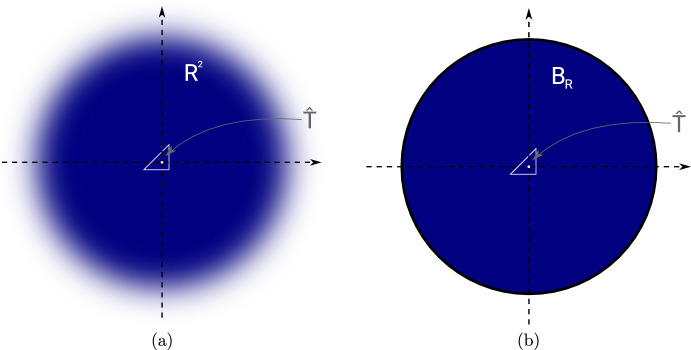
**a** Unbounded domain with reference triangle ${{\hat{T}}}$. **b** Truncated domain $B_R(0)$ with reference triangle ${{\hat{T}}}$

Passing to the limit $h \rightarrow 0$ yields an exterior problem on the unbounded domain, see Fig. 8(a) and again truncating this domain leads to the boundary value problem on the truncated domain $B_R(0)$ to find $W_{{{\hat{T}}}}[\lambda ^{\textrm{out}};{\varvec{e}}^{(k)}] \in H^1_0(B_R(0))$, $k=1,2$, as the unique solution to

57 $$\begin{aligned} \int \nolimits _{B_R(0)} \lambda ^{\textrm{out}}\nabla W_{{{\hat{T}}}}[\lambda ^{\textrm{out}};{\varvec{e}}^{(k)}] \cdot \nabla \psi \; \text{ d }x = - \int \nolimits _{{{\hat{T}}}} {\varvec{e}}^{(k)} \cdot \nabla \psi \; \text{ d }x \end{aligned}$$

for all $\psi \in H^1_0(B_R(0))$, see Fig. 8(b). Note that this problem differs from problem (48) only by a different scaling factor on the right hand side and by a homogeneous material distribution $\lambda ^{\textrm{out}}$.

In the case when $T_\ell$ is in an inhomogeneous region of the computational domain ${\textsf{D}}$ (i.e., not all neighbors of $T_\ell$ have the same material coefficient), we can follow the same averaging procedure with three sectors as in Sect. 4.2.2 and obtain the problem to find $W_{{{\hat{T}}}}[({\varvec{\lambda }}_\ell , \lambda _{T_\ell }^{S_1}, \lambda _{T_\ell }^{S_2}, \lambda _{T_\ell }^{S_3});{\varvec{e}}^{(k)}] \in H^1_0(B_R(0))$, $k=1,2$, as the unique solution to

58 $$\begin{aligned} \int \nolimits _{B_R(0)} \lambda _{{{\hat{T}}}} \nabla W_{{{\hat{T}}}}[({\varvec{\lambda }}_\ell , \lambda _{T_\ell }^{S_1}, \lambda _{T_\ell }^{S_2}, \lambda _{T_\ell }^{S_3});{\varvec{e}}^{(k)}] \cdot \nabla \psi \; \text{ d }x = - \int \nolimits _{{{\hat{T}}}} {\varvec{e}}^{(k)} \cdot \nabla \psi \; \text{ d }x \end{aligned}$$

where $\lambda _{{{\hat{T}}}}(x) = \chi _{{{\hat{T}}}}(x) {\varvec{\lambda }}_\ell + \sum _{j=1}^3 \chi _{S_j}(x) \lambda _{T_\ell }^{S_j}$, cf. also the material distribution in Fig. 6. Remark 9 concerning the numerical approximation of (57) and (58) with a mesh where the subdomain ${{\hat{T}}}$ of $B_R(0)$ is discretized by exactly one element remains valid. We define the $2\times 2$ matrix

59 $$\begin{aligned} \begin{aligned} {\varvec{\Gamma }}_{{{\hat{T}}},\ell } :=&{\varvec{\Gamma }}_{{{\hat{T}}}}[({\varvec{\lambda }}_\ell , \lambda _{T_\ell }^{S_1}, \lambda _{T_\ell }^{S_2}, \lambda _{T_\ell }^{S_3})] \\ :=&\left[ \frac{1}{{{\hat{T}}}} \int \nolimits _{{{\hat{T}}}}\nabla W_{{{\hat{T}}}}[({\varvec{\lambda }}_\ell , \lambda _{T_\ell }^{S_1}, \lambda _{T_\ell }^{S_2}, \lambda _{T_\ell }^{S_3});{\varvec{e}}^{(1)}] \text{ d }x \quad \frac{1}{{{\hat{T}}}} \int \nolimits _{{{\hat{T}}}}\nabla W_{{{\hat{T}}}}[({\varvec{\lambda }}_\ell , \lambda _{T_\ell }^{S_1}, \lambda _{T_\ell }^{S_2}, \lambda _{T_\ell }^{S_3});{\varvec{e}}^{(2)}] \text{ d }x\right] , \end{aligned} \end{aligned}$$

and remark that, in the same way as pointed out in Remark 10, we can also precompute the matrices ${\varvec{\Gamma }}_{{{\hat{T}}},\ell }$ for a four-dimensional array of values in an offline stage and interpolate them efficiently in the online stage. This way, we get the separable model

60 $$\begin{aligned} \hat{{\mathcal {J}}}_{\text {SMWapprox}}({\varvec{\eta }}) := {\mathcal {J}}({\varvec{\lambda }}) -\sum _{\ell =1}^m |T_\ell |({\varvec{\eta }}_\ell - {\varvec{\lambda }}_{\ell }) (\nabla u_h|_{T_\ell })^\top \left( {\varvec{I}}_2 - ({\varvec{\eta }}_\ell - {\varvec{\lambda }}_{\ell }) {\varvec{\Gamma }}_{{{\hat{T}}}, \ell }\right) ^{-1} \nabla u_h|_{T_\ell } \end{aligned}$$

as an approximation to the separable exact model (32).

## Relationships

We investigate the relationship between the model (52) of Sect. 4 that is motivated by the continuous concept of topological derivatives and the model (60) of Sect. 5 which is meant to approximate the Sherman–Morrison–Woodbury matrix identity model (32). We restrict our presentation to the case of homogeneous material distribution around the fixed element $T_\ell \in {\mathcal {T}}$.

We start by recalling the definitions of the discretized weak polarization matrix ${\mathcal {P}}_{{{\hat{T}}}, h}[\lambda ^{\textrm{out}}, \lambda ^{\textrm{in}}]$ (49) and the discretization of the matrix ${\varvec{\Gamma }}_{{{\hat{T}}}}$ (59) in the homogeneous setting,

61 $$\begin{aligned} {\mathcal {P}}_{{{\hat{T}}}, h}[\lambda ^{\textrm{out}}, \lambda ^{\textrm{in}}] =&\left[ \frac{1}{|{{\hat{T}}}|} \int \nolimits _{{{\hat{T}}}}\nabla K_{{{\hat{T}}},h}[\lambda ^{\textrm{out}}, \lambda ^{\textrm{in}};{\varvec{e}}^{(1)}]\text{ d }x \right.  &   \left. \frac{1}{|{{\hat{T}}}|} \int \nolimits _{{{\hat{T}}}} \nabla K_{{{\hat{T}}},h}[\lambda ^{\textrm{out}}, \lambda ^{\textrm{in}};{\varvec{e}}^{(2)}] \text{ d }x \right] \end{aligned}$$

62 $$\begin{aligned} {\varvec{\Gamma }}_{{{\hat{T}}}, h}[\lambda ^{\textrm{out}}] =&\left[ \frac{1}{{{\hat{T}}}} \int \nolimits _{{{\hat{T}}}}\nabla W_{{{\hat{T}}}, h}[\lambda ^{\textrm{out}};{\varvec{e}}^{(1)}] \text{ d }x \right.  &   \left. \frac{1}{{{\hat{T}}}} \int \nolimits _{{{\hat{T}}}}\nabla W_{{{\hat{T}}}, h}[\lambda ^{\textrm{out}};{\varvec{e}}^{(2)}] \text{ d }x\right] \end{aligned}$$

where $K_{{{\hat{T}}}, h}[\lambda ^{\textrm{out}}, \lambda ^{\textrm{in}}; {\varvec{e}}^{(k)}] \in V_{h}^R$ is the solution to (48) and $W_{{{\hat{T}}}, h}[\lambda ^{\textrm{out}}; {\varvec{e}}^{(k)}] \in V_{h}^R$ is the finite element approximation to (57), i.e.,

63 $$\begin{aligned} \int \nolimits _{B_R(0)}\lambda _{{{\hat{T}}} } \nabla K_{{{\hat{T}}},h}[\lambda ^{\textrm{out}}, \lambda ^{\textrm{in}};{\varvec{e}}^{(k)}] \cdot \nabla \psi _h \; \text{ d }x =&-(\lambda ^{\textrm{in}}- \lambda ^{\textrm{out}}) \int \nolimits _{{{\hat{T}}}} {\varvec{e}}^{(k)} \cdot \nabla \psi _h \; \text{ d }x \end{aligned}$$

64 $$\begin{aligned} \int \nolimits _{B_R(0)} \lambda ^{\textrm{out}}\nabla W_{{{\hat{T}}},h}[\lambda ^{\textrm{out}};{\varvec{e}}^{(k)}] \cdot \nabla \psi _h \; \text{ d }x =&- \int \nolimits _{{{\hat{T}}}} {\varvec{e}}^{(k)} \cdot \nabla \psi _h \; \text{ d }x \end{aligned}$$

for all $\psi _h \in V_{h}^R$. Here, recall that $\lambda _{{{\hat{T}}}}(x) = \chi _{{{\hat{T}}}}(x) \lambda ^{\textrm{in}}+ \chi _{B_R(0) {\setminus } {{\hat{T}}}}(x) \lambda ^{\textrm{out}}$.

We show the following relation between ${\mathcal {P}}_{{{\hat{T}}}, h}[\lambda ^{\textrm{out}}, \lambda ^{\textrm{in}}]$ and ${\varvec{\Gamma }}_{{{\hat{T}}}, h}[\lambda ^{\textrm{out}}]$:

### Theorem 9

It holds that

65 $$\begin{aligned} {\mathcal {P}}_{{{\hat{T}}}, h}[\lambda ^{\textrm{out}}, \lambda ^{\textrm{in}}] = (\lambda ^{\textrm{in}}- \lambda ^{\textrm{out}}) {\varvec{\Gamma }}_{{{\hat{T}}}, h}[\lambda ^{\textrm{out}}] \left( {\varvec{I}}_2 - (\lambda ^{\textrm{in}}- \lambda ^{\textrm{out}}) {\varvec{\Gamma }}_{{{\hat{T}}}, h}[\lambda ^{\textrm{out}}] \right) ^{-1} \end{aligned}$$

and further

66 $$\begin{aligned} {\varvec{I}}_2 +{\mathcal {P}}_{{{\hat{T}}}, h}[\lambda ^{\textrm{out}}, \lambda ^{\textrm{in}}] = ({\varvec{I}}_2 - (\lambda ^{\textrm{in}}- \lambda ^{\textrm{out}}) {\varvec{\Gamma }}_{{{\hat{T}}}, h}[\lambda ^{\textrm{out}}] )^{-1}. \end{aligned}$$

### Proof

Recall that we use piecewise linear and globally continuous finite elements on a triangular mesh of *M* elements of $B_R(0)$ where, according to Remark 9, the subdomain ${{\hat{T}}}$ is resolved by exactly one triangle of the mesh. Let now the finite element stiffness matrix of (63) be denoted by ${{\tilde{{\varvec{A}}}}}$ and the one of (64) by ${\varvec{A}}$ where we use the same mesh and finite element space for both equations. Let ${\hat{\ell }} \in \{1, \dots , M\}$ be the element index corresponding to the triangle ${{\hat{T}}}$. Note that the material distribution in (63) differs from that in (64) only in element ${\hat{\ell }}$ and we have ${\varvec{A}}= \sum _{k=1}^M \lambda ^{\textrm{out}}B_k B_k^\top$ and

$$\begin{aligned} {{\tilde{{\varvec{A}}}}} = {\varvec{A}}+ (\lambda ^{\textrm{in}}- \lambda ^{\textrm{out}}) {\varvec{B}_{{\hat{\ell }}}}{\varvec{B}_{{\hat{\ell }}}}^\top , \end{aligned}$$

thus, an application of the Sherman–Morrison–Woodbury formula of Lemma 4 yields

67 $$\begin{aligned} {{\tilde{{\varvec{A}}}}}^{-1} = {\varvec{A}}^{-1} - (\lambda ^{\textrm{in}}- \lambda ^{\textrm{out}}) {\varvec{A}}^{-1} {\varvec{B}_{{\hat{\ell }}}}\left( {\varvec{I}}_2 + (\lambda ^{\textrm{in}}- \lambda ^{\textrm{out}}) {\varvec{B}_{{\hat{\ell }}}}^\top {\varvec{A}}^{-1} {\varvec{B}_{{\hat{\ell }}}}\right) ^{-1} {\varvec{B}_{{\hat{\ell }}}}^\top {\varvec{A}}^{-1}. \end{aligned}$$

On the other hand, we know from Lemma 8 that for the chosen piecewise linear finite elements where ${{\hat{T}}}$ is resolved by only one triangle (i.e. $\nabla W_{{{\hat{T}}},h}[\lambda ^{\textrm{out}}; {\varvec{e}}^{(k)}](x)$, $\nabla K_{{{\hat{T}}}, h}[\lambda ^{\textrm{out}}, \lambda ^{\textrm{in}};{\varvec{e}}^{(k)}](x)$ are constant on ${{\hat{T}}}$), we have

$$\begin{aligned}&(\lambda ^{\textrm{in}}- \lambda ^{\textrm{out}}) {\varvec{\Gamma }}_{{{\hat{T}}},h}[\lambda ^{\textrm{out}}] = - (\lambda ^{\textrm{in}}- \lambda ^{\textrm{out}}) {\varvec{B}_{{\hat{\ell }}}}^\top {\varvec{A}}^{-1} {\varvec{B}_{{\hat{\ell }}}}\\&\quad \text{ and } \quad {\mathcal {P}}_{{{\hat{T}}},h}[\lambda ^{\textrm{out}}, \lambda ^{\textrm{in}}]= - (\lambda ^{\textrm{in}}- \lambda ^{\textrm{out}}) {\varvec{B}_{{\hat{\ell }}}}^\top {{\tilde{{\varvec{A}}}}}^{-1} {\varvec{B}_{{\hat{\ell }}}}. \end{aligned}$$

Thus, denoting ${\varvec{\Gamma }}_{{{\hat{T}}}, h}^\lambda := (\lambda ^{\textrm{in}}-\lambda ^{\textrm{out}}){\varvec{\Gamma }}_{{{\hat{T}}}, h}[\lambda ^{\textrm{out}}]$ and plugging in (67) yields

$$\begin{aligned} {\mathcal {P}}_{{{\hat{T}}},h}[\lambda ^{\textrm{out}}, \lambda ^{\textrm{in}}] =&{\varvec{\Gamma }}_{{{\hat{T}}}, h}^\lambda + {\varvec{\Gamma }}_{{{\hat{T}}}, h}^\lambda ({\varvec{I}}_2 - {\varvec{\Gamma }}_{{{\hat{T}}}, h}^\lambda )^{-1} {\varvec{\Gamma }}_{{{\hat{T}}}, h}^\lambda \\ =&{\varvec{\Gamma }}_{{{\hat{T}}}, h}^\lambda ({\varvec{I}}_2 +({\varvec{I}}_2 - {\varvec{\Gamma }}_{{{\hat{T}}}, h}^\lambda )^{-1} {\varvec{\Gamma }}_{{{\hat{T}}}, h}^\lambda ) \\ =&{\varvec{\Gamma }}_{{{\hat{T}}}, h}^\lambda ({\varvec{I}}_2 - {\varvec{\Gamma }}_{{{\hat{T}}}, h}^\lambda )^{-1} \end{aligned}$$

where we used the identity $({\varvec{I}}- {\varvec{B}})^{-1} = {\varvec{I}}+ ({\varvec{I}}-{\varvec{B}})^{-1} {\varvec{B}}$ for any matrix ${\varvec{B}}$ such that ${\varvec{I}}-{\varvec{B}}$ is invertible in the last step. This proves (65). In order to see (66) note that, by (65), it holds ${\varvec{I}}_2 + {\mathcal {P}}_{{{\hat{T}}},h}[\lambda ^{\textrm{out}}, \lambda ^{\textrm{in}}] = {\varvec{I}}_2 + {\varvec{\Gamma }}_{{{\hat{T}}}, h}^\lambda ({\varvec{I}}_2 - {\varvec{\Gamma }}_{{{\hat{T}}}, h}^\lambda )^{-1} = ({\varvec{I}}_2 - {\varvec{\Gamma }}_{{{\hat{T}}}, h}^\lambda + {\varvec{\Gamma }}_{{{\hat{T}}}, h}^\lambda ) ({\varvec{I}}- {\varvec{\Gamma }}_{{{\hat{T}}}, h}^\lambda )^{-1} = ({\varvec{I}}- {\varvec{\Gamma }}_{{{\hat{T}}}, h}^\lambda )^{-1}$.$\square$

### Corollary 10

From Theorem 9 it follows immediately that the two models $\hat{{\mathcal {J}}}_{\text {TDnum}}$ defined in (52) and $\hat{{\mathcal {J}}}_{\text {SMWapprox}}$ defined in (60) coincide.

### Remark 11

We remark that the same proof can be conducted in the case of inhomogeneous material around the element of interest and the statements of Theorem 9 and Corollary 10 remain valid also in that case.

At the first glance, Theorem 9 and Corollary 10 seem very surprising since they state that the model that is based on the continuous concept of topological derivatives brought to a discrete setting coincides with a model that is based on a certain approximation of a term generated by the purely algebraic Sherman–Morrison–Woodbury matrix identity. This resemblance, however, has been identified in [32] on the purely continuous setting for the case of elliptic inclusions.

### Lemma 11

([32]) Assume that $\omega$ is an ellipse. Then

68 $$\begin{aligned} {\mathcal {P}}_\omega [\lambda ^{\textrm{out}}, \lambda ^{\textrm{in}}] = -(\lambda ^{\textrm{in}}- \lambda ^{\textrm{out}}) \left( {\varvec{I}}_2 + (\lambda ^{\textrm{in}}- \lambda ^{\textrm{out}}) {\varvec{\Psi }}[\lambda ^{\textrm{out}}] \right) ^{-1} {\varvec{\Psi }}[\lambda ^{\textrm{out}}] \end{aligned}$$

where ${\varvec{\Psi }}[\lambda ^{\textrm{out}}] \in {\mathbb {R}}^{2\times 2}$ is given by

69 $$\begin{aligned} {\varvec{\Psi }}[\lambda ^{\textrm{out}}]_{i,j} = - \left( \int \nolimits _{\partial \omega } n(x) \nabla _x \Phi [\lambda ^{\textrm{out}}](x)^\top \; \text{ d }s_x \right) _{i,j} = - \int \nolimits _{\partial \omega } n_i \partial _{x_j} \Phi [\lambda ^{\textrm{out}}](x) \; \text{ d }s_x \end{aligned}$$

with the fundamental solution $\Phi [\lambda ^{\textrm{out}}]$ of the operator $u \mapsto - \text{ div }(\lambda ^{\textrm{out}}\nabla u)$, i.e., $\Phi [\lambda ^{\textrm{out}}](x) = - 1/ (2 \pi \lambda ^{\textrm{out}}) \text{ ln }(|x|)$.

### Proof

This follows straightforwardly from [32, Sec. 8] by restricting the (vector-valued) elasticity problem treated there to the scalar Laplace-type problem considered here. $\square$

From Lemma 11, it follows in the same way as in the proof of Theorem 9 that

70 $$\begin{aligned} {\varvec{I}}_2 + {\mathcal {P}}_\omega [\lambda ^{\textrm{out}}, \lambda ^{\textrm{in}}] = ({\varvec{I}}_2 + (\lambda ^{\textrm{in}}- \lambda ^{\textrm{out}}) {\varvec{\Psi }}[\lambda ^{\textrm{out}}] )^{-1}, \end{aligned}$$

and thus, from (21), we get the alternative representation of the topological derivative for elliptic inclusion shapes $\omega$

71 $$\begin{aligned} d {\mathcal {J}}[\lambda ^{\textrm{out}}, \lambda ^{\textrm{in}}](\Omega )(z, \omega ) = (\lambda ^{\textrm{in}}- \lambda ^{\textrm{out}}) \nabla u(z)^\top ({\varvec{I}}_2 + (\lambda ^{\textrm{in}}- \lambda ^{\textrm{out}}) {\varvec{\Psi }}[\lambda ^{\textrm{out}}] )^{-1} \nabla p(z). \end{aligned}$$

Thus, the matrix ${\varvec{\Gamma }}_{{{\hat{T}}}, h}[\lambda ^{\textrm{out}}]$ used in $\hat{{\mathcal {J}}}_{\text {SMWapprox}}$ can also be seen as an approximation to the negative fundamental matrix $-{\varvec{\Psi }}[\lambda ^{\textrm{out}}]$. Finally, note that the proof of Lemma 11 in [32] is not valid for triangular inclusion shapes, however, at the discrete level, relation (65) still holds. Furthermore, note that the matrix ${\varvec{\Psi }}[\lambda ^{\textrm{out}}]$ is symmetric such that the right hand side of (68) can also be written as $-(\lambda ^{\textrm{in}}- \lambda ^{\textrm{out}}) {\varvec{\Psi }}[\lambda ^{\textrm{out}}]\left( {\varvec{I}}_2 + (\lambda ^{\textrm{in}}- \lambda ^{\textrm{out}}) {\varvec{\Psi }}[\lambda ^{\textrm{out}}] \right) ^{-1}$ which coincides with the structure of (65).

## Numerical Experiments

In this section, we examine the models introduced in Sects. 4 and 5 and compare them to the exact solution as well as the diagonal approximation model introduced in Sect. 3. Since we noted in Sect. 6 that model (52) of Sect. 4 and model (60) of Sect. 5 coincide, we will here only consider the latter model. We remark that coincidence of the two models was observed also in all numerical examples.

All numerical results are illustrated for the model problem introduced in Sect. 2.1 with the two-dimensional computational domain ${\textsf{D}}= (0,1)^2$ with Dirichlet and Neumann boundaries $\Gamma _D = \{(0,y), y \in (0,1)\}\cup \{(x,0), x \in (0,1)\}$, $\Gamma _N = \partial {\textsf{D}}{\setminus } \Gamma _N$ with corresponding data $g_D = 0$, $g_N(x_1, x_2) = x_1 x_2$ and the constant source term $f(x_1, x_2) = 1$. The material coefficient $\lambda (x)$ will vary between the values ${\underline{\lambda }}=1$ and ${\overline{\lambda }}=1000$.

We begin by considering a homogeneous setting.

### Homogeneous Material Distribution

**Fig. 9:**
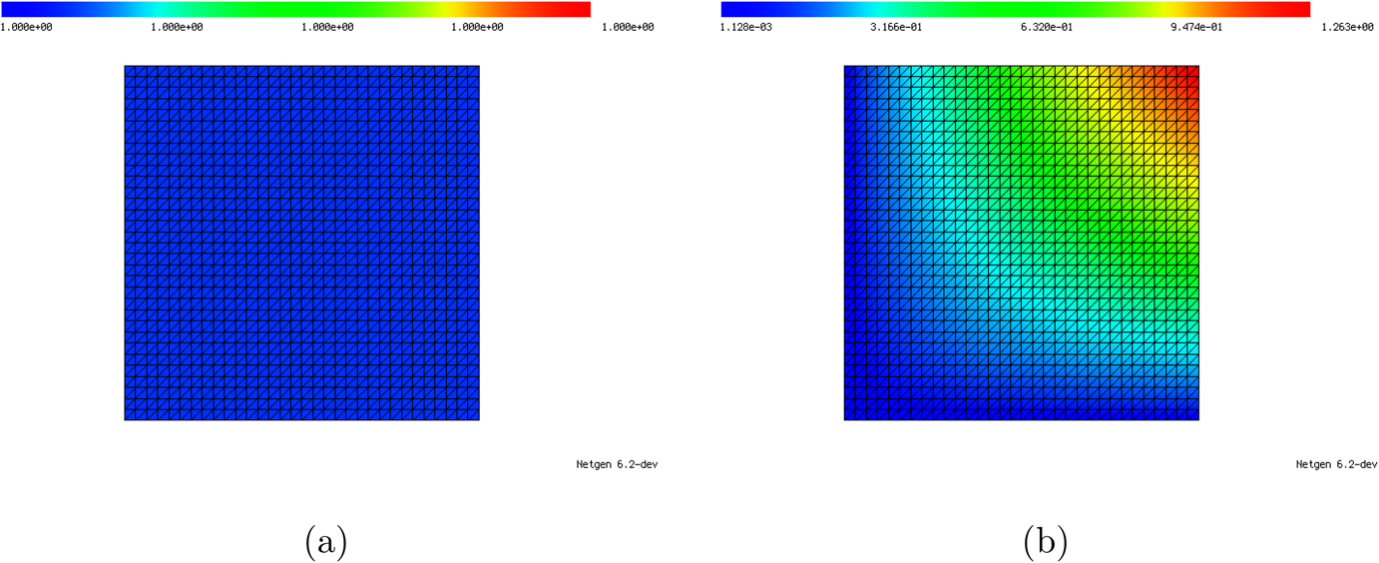
**a** Material coefficient $\lambda (x)$ for homogeneous setting. **b** Finite element solution $u_h$ of problem (5b) with data specified in Sect. 7 for homogeneous material distribution

Here, we consider a constant material distribution, i.e., $\lambda _\Omega (x) = \lambda ^{\textrm{out}}$ for all $x \in {\textsf{D}}$, which corresponds to setting $\Omega = \emptyset$ for some value $\lambda ^{\textrm{out}}$ in the setting of Sect. 2.1.1. See also Fig. 9 for plots of the material distribution and the finite element solution on a mesh with $n=1089$ nodes and $m=2048$ elements.

#### Numerical Comparison of Different Models for Fixed Element

**Fig. 10:**
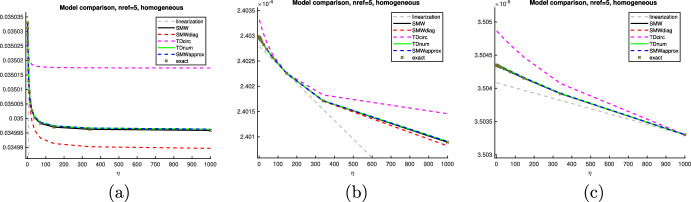
Comparison of different models for homogeneous setting with background material **a** $\lambda ^{\textrm{out}}= 1$, **b** $\lambda ^{\textrm{out}}\approx 145.834$, **c** $\lambda ^{\textrm{out}}= 1000$ as functions of the perturbed material coefficient in a fixed element $T_\ell$

We fix the interior element $T_\ell \in {\mathcal {T}}$ as that element of type 1 (cf. Fig. 1) that has the point (0.5, 0.25) as its bottom right vertex and compare the different models we introduced in the previous sections for the case where the homogeneous material distribution is perturbed only in that one element $T_\ell$, i.e., ${\varvec{\eta }}= {\varvec{\lambda }}+ (\eta - {\varvec{\lambda }}_\ell ){\varvec{e}}^{(\ell )}$. Figure 10 shows the different models as functions of the perturbed value $\eta \in [{\underline{\lambda }}, {\overline{\lambda }}] = [1, 1000]$ for three different background material values $\lambda ^{\textrm{out}}= 1$, $\lambda ^{\textrm{out}}\approx 145.834$, $\lambda ^{\textrm{out}}= 1000$ (cf. Remark 12). In Fig. 10 we can see the exact solution ${\mathcal {J}}({\varvec{\eta }})$ (where the perturbed stiffness matrix is inverted) for certain values of $\eta$ along with the exact Sherman–Morrison–Woodbury model $\hat{{\mathcal {J}}}_{\text {SMW}}$ (32) which shows, as expected, perfect coincidence. Moreover, we can see the diagonal approximation of the Sherman–Morrison–Woodbury model $\hat{{\mathcal {J}}}_{\text {SMWdiag}}$ (35) which shows a certain error, but can be evaluated more efficiently. The models $\hat{{\mathcal {J}}}_{\text {TDnum}}$ (52) and $\hat{{\mathcal {J}}}_{\text {SMWapprox}}$ (60) can be seen to match exactly, as was predicted by Theorem 9 and Corollary 10. Moreover, it can be seen from Fig. 10 that these two models approximate the exact solution extraordinarily well while being cheap to evaluate during the online stage (after precomputations have been done in an offline stage, cf. Remark 10). For comparison, we also included the linearization ${\mathcal {J}}({\varvec{\eta }}) \approx {\mathcal {J}}({\varvec{\lambda }}) - |T_\ell | (\eta - {\varvec{\lambda }}_\ell ) |\nabla u_h|_{T_\ell }|^2$ and the topological derivative model when the analytical formula for the topological derivative of circular inclusions (22) is used. It can be seen that the linearization model is far away from the true solution. But also the latter model shows a significant error which confirms the necessity to account for the triangular inclusion shape as it was done in Sect. 4.

In order to quantify these errors, let us define the relative error measure of a model $\hat{{\mathcal {J}}}$ in an element $T_\ell$ for a given material distribution ${\varvec{\lambda }}$ by

72 $$\begin{aligned} \delta \hat{{\mathcal {J}}}[T_\ell ] := \frac{1}{\Delta {\mathcal {J}}[T_\ell ]} \; \underset{\eta \in [{\underline{\lambda }}, {\overline{\lambda }}]}{\text{ max } } \left|\hat{{\mathcal {J}}}\left( {\varvec{\lambda }}+ (\eta - {\varvec{\lambda }}_\ell ){\varvec{e}}^{(\ell )}\right) -{\mathcal {J}}\left( {\varvec{\lambda }}+ (\eta - {\varvec{\lambda }}_\ell ){\varvec{e}}^{(\ell )}\right) \right|\end{aligned}$$

where $\Delta {\mathcal {J}}[T_\ell ] = \text{ max}_{t\in ({\underline{\lambda }}, {\overline{\lambda }})} {\mathcal {J}}({\varvec{\lambda }}+ (t - {\varvec{\lambda }}_\ell ){\varvec{e}}^{(\ell )}) - \text{ min } _{t\in ({\underline{\lambda }}, {\overline{\lambda }})} {\mathcal {J}}({\varvec{\lambda }}+ (t - {\varvec{\lambda }}_\ell ){\varvec{e}}^{(\ell )})$ is the difference of maximal and minimal values of the exact model in $T_\ell$. Thus, $\delta \hat{{\mathcal {J}}}[T_\ell ]$ measures the maximum relative error of a model $\hat{{\mathcal {J}}}$ in element $T_\ell$ relative to the variation of the exact cost function ${\mathcal {J}}$. The relative errors according to (72) for the three expansion points $\lambda (x) = \lambda ^{\textrm{out}}$ investigated in Fig. 10 are as follows: For the linearization model the errors are as high as (20 954$\%$, 85.61$\%$, 25.21$\%$), for the model $\hat{{\mathcal {J}}}_{\text {SMWdiag}}$ they are (16.65$\%$, 3.41$\%$, 0.99$\%$), for $\hat{{\mathcal {J}}}_{\text {TDcirc}}$ we have (57.93$\%$, 27.23$\%$, 49.43$\%$) and for the coinciding models $\hat{{\mathcal {J}}}_{\text {TDnum}}$ and $\hat{{\mathcal {J}}}_{\text {SMWapprox}}$ we have the values (1.18$\%$, 0.74$\%$, 1.46$\%$).

##### Remark 12

**Fig. 11:**
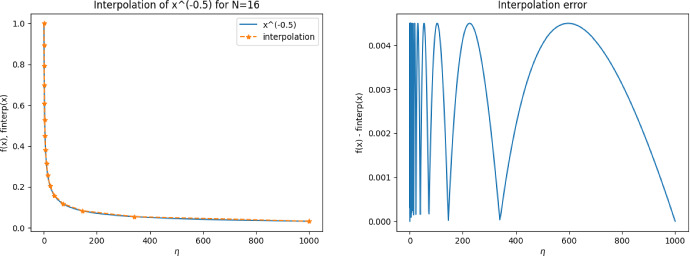
The interpolation nodes $\eta ^{(k)}$, $k=1, \dots , 16$ are chosen in such a way that the maximum interpolation error between the function $x^{-0.5}$ and its piecewise linear interpolant attains the same value in each interval $(\eta ^{(k)}, \eta ^{(k+1)})$

In Fig. 10, the models as well as the exact solution were evaluated at certain perturbation values $\eta ^{(k)} \in [1, 1000]$, $k=1, \dots , 16$. These values have been chosen in the following way: It was observed numerically that the exact solution in Fig. 10 behaves similarly to $a+b \eta ^{-0.5}$ for some constants *a*, *b*. Based on this observation, the points $\eta ^{(k)}$ were chosen in such a way that, using these points as interpolation nodes, a piecewise linear interpolation of $a+b \eta ^{-0.5}$ yields an equilibrated error, see Fig. 11. This was achieved by solving a system of nonlinear equations ensuring that the maximum interpolation error between any two neighboring nodes is equal. The values for $N=16$ points are given in Table 1. While these points here are used solely for visualization purposes, their role will become more important in Sect. 7.2 to decide for which values of the material coefficient the (computationally expensive) precomputation should be carried out.

##### Remark 13

Also the choice of the Hölder parameter $\alpha =-0.5$ in (50) was motivated by the observation that the exact solution in Fig. 10a behaves roughly like $a+b \eta ^{-0.5} =: f(\eta )$. Thus, an effective value for $\lambda$ can be obtained by the relation $f(\lambda ) = \sum w_i f(\lambda _i)$ for given values $\lambda _i$ with corresponding weights $w_i$ such that $\sum w_i = 1$, which, by inverting *f*, results in the chosen average value (50).

**Table 1:** Values $\eta ^{(k)}$, $k=1,\dots , 16$, used for visualization and precomputation

1	1.252	1.590	2.050	2.688	3.596	4.921	6.917
10.035	15.127	23.901	40.072	72.563	145.834	340.187	1000

#### Numerical Comparison of Different Models on Whole Domain

In Fig. 10, we compared several different models for a fixed triangle in the mesh when the material perturbation parameter is varied between ${\underline{\lambda }}=1$ and ${\overline{\lambda }}=1000$. Next, we investigate the maximum relative error $\delta \hat{{\mathcal {J}}}$ as defined in (72) of a model $\hat{{\mathcal {J}}}$ as a function of the position in space.

**Fig. 12:**
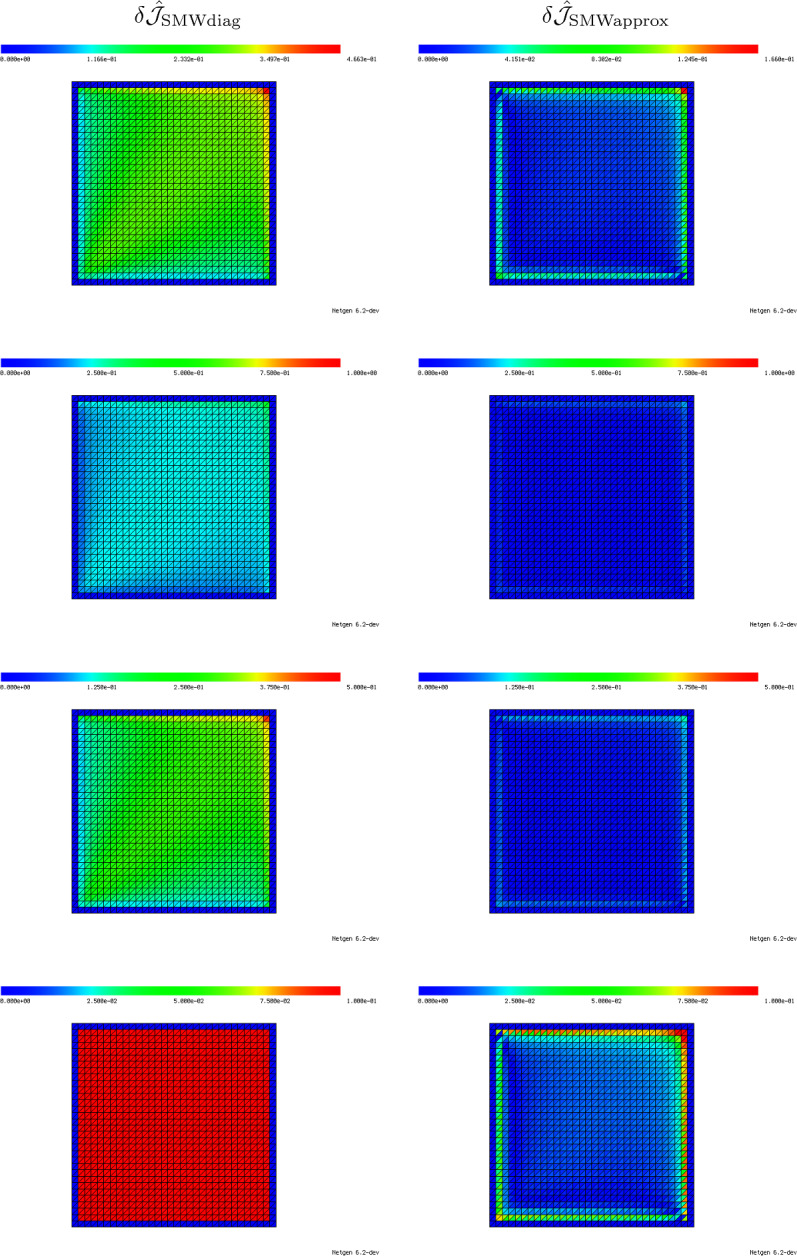
Comparison of relative errors $\delta \hat{{\mathcal {J}}}[T_\ell ]$ according to (72) for models $\hat{{\mathcal {J}}}_{\text {SMWdiag}}$ (35) in left column and $\hat{{\mathcal {J}}}_{\text {SMWapprox}}$ (60) ($=\hat{{\mathcal {J}}}_{\text {TDnum}}$ (52)) in right column for all interior elements $T_\ell$ in homogeneous setting. First line shows color plot according to their respective maximum errors. Second to fourth line show threshold for maximum relative error at $100 \%$, $50\%$ and $10\%$, respectively. Errors in elements touching the boundary are not computed

Given the findings of Fig. 10, we will focus on the two approximate Sherman–Morrison–Woodbury models $\hat{{\mathcal {J}}}_{\text {SMWdiag}}$ and $\hat{{\mathcal {J}}}_{\text {SMWapprox}}$ (recall that $\hat{{\mathcal {J}}}_{\text {TDnum}}$ coincides with $\hat{{\mathcal {J}}}_{\text {SMWapprox}}$). In Fig. 12, we plot the maximum relative errors (72) for these two models for all interior elements of the computational domain for the homogeneous material distribution $\lambda (x) = \lambda ^{\textrm{out}}= 1$, i.e., we are in the setting of Fig. 10a. Elements touching the boundary are discussed separately in Sect. 7.3.1. Here, we can see that the maximum relative error of the model $\hat{{\mathcal {J}}}_{\text {SMWdiag}}$ is around $47\%$ whereas it is around $17\%$ for $\hat{{\mathcal {J}}}_{\text {SMWapprox}}$. The four different rows of Fig. 12 show different threshold values for the relative errors in the color bars. Moreover, it can be seen from the right column in Fig. 12 that the model $\hat{{\mathcal {J}}}_{\text {SMWapprox}}$ behaves particularly well in the center of the homogeneous domain and that the error increases slightly the closer one gets to a boundary. This was to be expected since the idea of the model $\hat{{\mathcal {J}}}_{\text {SMWapprox}}$ in Sect. 5 (and equivalently model $\hat{{\mathcal {J}}}_{\text {TDnum}}$ of Sect. 2.2) was to zoom in locally around the fixed element $T_\ell$ and assume that all boundaries are sufficiently far away, cf. Figs. 7 and 8. Nevertheless, the maximum error attained by $\hat{{\mathcal {J}}}_{\text {SMWapprox}}$ is still significantly smaller than that of the model $\hat{{\mathcal {J}}}_{\text {SMWdiag}}$.

#### Towards Topology Optimization Using Approximate Models

We want to illustrate the potential of the introduced models $\hat{{\mathcal {J}}}_{\text {SMWdiag}}$ and $\hat{{\mathcal {J}}}_{\text {SMWapprox}}$ (which coincides with $\hat{{\mathcal {J}}}_{\text {TDnum}}$) in the course of a binary topology optimization algorithm. Here, we simply decide for each element $T_\ell$ if it should be occupied by $\lambda ^{\textrm{out}}= 1$ or $\lambda ^{\textrm{in}}= 1000$ based on the values of a model $\hat{{\mathcal {J}}}({\varvec{\lambda }}+ (\eta -{\varvec{\lambda }}_\ell ){\varvec{e}}^{(\ell )})$ at $\eta =\lambda ^{\textrm{out}}$ and $\eta = \lambda ^{\textrm{in}}$, i.e., we do not allow for intermediate material values. Here, we again start out from the homogeneous design where ${\varvec{\lambda }}\in {\mathbb {R}}^m$ is the constant one vector. As a reference, we consider the separable exact model $\hat{{\mathcal {J}}}_{\text {SMW}}$ (32). Due to this model’s properties, the material distribution obtained by the mentioned procedure is a local minimum, which cannot be improved by switching the state of only one element. Note that this is a stronger notion of optimality than a design being solely a stationary point of the relaxed optimization problem. From a theoretical point of view it is not entirely clear that using approximations of exact separable models such as $\hat{{\mathcal {J}}}_{\text {SMWdiag}}$ the same effect can be achieved. However, already in [19] it was reported that the SGP concept combined with an approximation of the $\hat{{\mathcal {J}}}_{\text {SMWdiag}}$ type lead to a much better local minimizer for a binary topology optimization problem than the MMA method utilizing convex separable approximations. Here, we investigate this effect in more detail using a selection of the previously suggested models. In addition, we also make a comparison with an MMA model

73 $$\begin{aligned} \hat{{\mathcal {J}}}_{\text {MMA}}({\varvec{\eta }})&= {\mathcal {J}}({\varvec{\lambda }}) - \sum _{\ell =1}^m |T_\ell | ({\varvec{\eta }}_\ell - {\varvec{\lambda }}_\ell ) (\nabla u_h|_{T_\ell })^\top \left( {\varvec{I}}_2 -\frac{{\varvec{\eta }}_\ell - {\varvec{\lambda }}_\ell }{L-{\varvec{\lambda }}_\ell } {\varvec{I}}_2 \right) ^{-1}\nabla u_h|_{T_\ell }\nonumber \\&= {\mathcal {J}}({\varvec{\lambda }}) - \sum _{\ell =1}^m |T_\ell | | \nabla u_h|_{T_\ell } |^2 \frac{({\varvec{\eta }}_\ell - {\varvec{\lambda }}_\ell )({\varvec{\lambda }}_\ell - L)}{ ({\varvec{\eta }}_\ell - L)}. \end{aligned}$$

Here *L* plays the role of a vertical asymptote, which is chosen individually for each element by a heuristic update scheme in the original MMA method, see [17]. As we consider only a single update step here, the heuristic for the choice of *L* can not be applied. Instead we test three different constant choices of the asymptote, $L=0$, $L=-5$, $L=-10$. Note that (73) can be obtained from (34) by replacing ${\varvec{\Gamma }}^{(\ell )}$ by $\frac{1}{L-{\varvec{\lambda }}_\ell } {\varvec{I}}_2$.

**Fig. 13:**
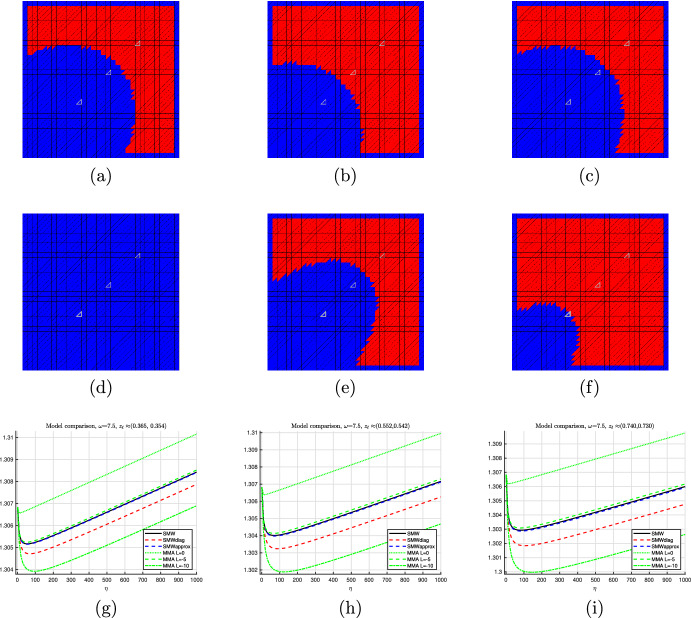
Top and central row: Material distribution after one step of binary topology optimization for (74) with $\omega = 7.5$ starting out from homogeneous material $\lambda (x) = 1$ (see Fig. 9a) when using **a** exact compliance model $\hat{{\mathcal {J}}}_{\text {SMW}}$, **b** diagonal approximation to Sherman–Morrison–Woodbury model $\hat{{\mathcal {J}}}_{\text {SMWdiag}}$, **c** approximate compliance model $\hat{{\mathcal {J}}}_{\text {SMWapprox}}$, **d** MMA model with $L=0$, **e** MMA model with $L=-5$, **f** MMA model with $L=-10$. Bottom row: Illustration of local models in three triangles marked in top row from bottom left (**g**) to top right (**i**)

Since, as it is well-known, the optimum material design for compliance minimization without limitation on the volume is the full design, we here include a simple volume penalization in the cost function and use the augmented cost function

74 $$\begin{aligned} {\mathcal {L}}({\varvec{\lambda }}):= {\mathcal {J}}({\varvec{\lambda }}) + \omega \text {Vol}({\varvec{\lambda }}) \end{aligned}$$

with a fixed weight $\omega = 7.5$ and the volume of the strong material $\text {Vol}({\varvec{\lambda }}) = \sum _{\ell =1}^m |T_\ell | ({\varvec{\lambda }}_\ell - \lambda ^{\textrm{out}}) / (\lambda ^{\textrm{in}}- \lambda ^{\textrm{out}})$. Note that $\text {Vol}({\varvec{\lambda }})$ itself is a separable function which can be dealt with without approximation error. Figure 13 shows the designs obtained after one step of the procedure mentioned above when using (a) the exact (but expensive) model $\hat{{\mathcal {J}}}_{\text {SMW}}$, (b) the diagonal approximation $\hat{{\mathcal {J}}}_{\text {SMWdiag}}$, (c) the proposed model $\hat{{\mathcal {J}}}_{\text {SMWapprox}}$ and (d)–(f) the MMA model with $L=0$, $L=-5$ and $L=-10$. Comparing pictures (b) and (c) to (a), we see that the error in the design produced by the model in (c) is almost zero, whereas it is a bit larger for the diagonal approximation model in (b). The performance of the MMA model here depends heavily on the choice of the parameter *L*. For illustration of the method, we also plotted the curves corresponding to the exact and the two mentioned approximate models in three fixed elements. Figure 13g shows that, in the leftmost of the three highlighted elements in (a)–(f), the value of all six considered models at $\lambda ^{\textrm{in}}=1000$ is higher than at $\lambda ^{\textrm{out}}= 1$, thus making a switching of the material from $\lambda ^{\textrm{out}}$ to $\lambda ^{\textrm{in}}$ unattractive. In the same way, in the rightmost of the three marked elements, all models except for MMA with $L=0$ show smaller values at $\lambda ^{\textrm{in}}$ than at $\lambda ^{\textrm{out}}$, thus suggesting switching the material to decrease the cost function, see Fig. 13i. In the central one out of these three elements, however, the diagonal approximation model $\hat{{\mathcal {J}}}_{\text {SMWdiag}}$ suggests to switch the material since its value is smaller at $\lambda ^{\textrm{in}}$ than at $\lambda ^{\textrm{out}}$, whereas the exact model as well as the proposed approximation $\hat{{\mathcal {J}}}_{\text {SMWapprox}}$ suggest not to switch it, see Fig. 13h. The MMA model shows good behavior for the choice $L=-5$, but large errors for the choices $L=0$ and $L=-10$. Finally, we remark that, when comparing with the exact model $\hat{{\mathcal {J}}}_{\text {SMW}}$, for $\hat{{\mathcal {J}}}_{\text {SMWdiag}}$ the wrong decision was taken for 280 out of 1800 interior elements whereas this was the case only for 19 elements in the case of $\hat{{\mathcal {J}}}_{\text {SMWapprox}}$. For the MMA model with $L=0$, $L=-5$, $L=-10$, the numbers of wrongly switched elements were 930, 102 and 586 elements, respectively.

### Inhomogeneous Material Distribution

**Fig. 14:**
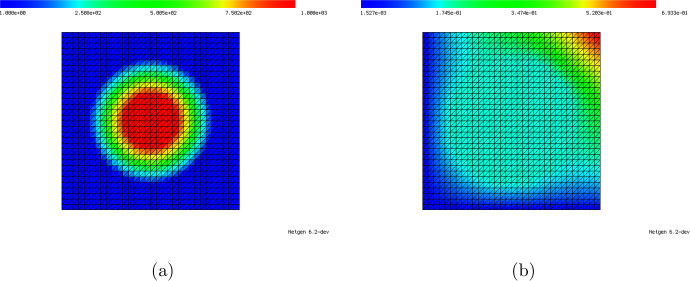
**a** Material coefficient $\lambda$ for inhomogeneous setting. **b** Finite element solution $u_h$ of problem (5b) with data specified in Sect. 7 for inhomogeneous material distribution

Next we consider a numerical example with an inhomogeneous material distribution as it may appear in the course of a density-based topology optimization algorithm, which is the motivation for this work. We consider a material coefficient $\lambda (x)$ that continuously varies between ${\underline{\lambda }}= 1$ and ${\overline{\lambda }}= 1000$ as

$$\begin{aligned} \lambda (x) = {\left\{ \begin{array}{ll} {\underline{\lambda }}, &  |x-m| \ge r_2, \\ {\underline{\lambda }}+\frac{|x-m|-r_1}{r_2 - r_1}({\overline{\lambda }}- {\underline{\lambda }}), &  |x-m| \in ( r_1, r_2), \\ {\overline{\lambda }}, &  |x-m| \le r_1, \end{array}\right. } \end{aligned}$$

with $r_1 = 0.15$, $r_2 = 0.35$ and $m = (0.5, 0.5)^\top$. The material distribution and the corresponding finite element solution of (5b) with the data defined in the beginning of Sect. 7 are depicted in Fig. 14.

#### Numerical Evaluation of $\hat{{\mathcal {J}}}_{\text {SMWapprox}}$ in Inhomogeneous Setting

In order to evaluate $\hat{{\mathcal {J}}}_{\text {SMWapprox}}$ in inhomogeneous regions of the computational domain, recall that model (60) involves the matrix ${\varvec{\Gamma }}_{{{\hat{T}}}, \ell } = {\varvec{\Gamma }}_{{{\hat{T}}}}[({\varvec{\lambda }}_\ell , \lambda _{T_\ell }^{S_1}, \lambda _{T_\ell }^{S_2}, \lambda _{T_\ell }^{S_3})]$ defined in (59) where $\lambda _{T_\ell }^{S_1}, \lambda _{T_\ell }^{S_2}, \lambda _{T_\ell }^{S_3}$ are averaged material coefficients according to (50), see also Fig. 6. The matrix ${\varvec{\Gamma }}_{{{\hat{T}}}, \ell }$, in turn, is based on the solution to the truncated transmission problem (58) for the given averaged material values. Thus, in order to evaluate $\hat{{\mathcal {J}}}_{\text {SMWapprox}}$, that exterior problem would have to be solved for the averaged material values of every single element, which would make the model computationally intractable.

For that reason, we introduce another approximation step: We precompute the matrix ${\varvec{\Gamma }}_{{{\hat{T}}}}[({\varvec{\lambda }}_\ell , \lambda _{T_\ell }^{S_1}, \lambda _{T_\ell }^{S_2}, \lambda _{T_\ell }^{S_3})]$ for a large discrete set of combinations of material coefficients in an offline stage. More precisely, we precompute the matrix ${\varvec{\Gamma }}_{{{\hat{T}}}}[({\varvec{\lambda }}_\ell , \lambda _{T_\ell }^{S_1}, \lambda _{T_\ell }^{S_2}, \lambda _{T_\ell }^{S_3})]$ for all combinations of tuples $({\varvec{\lambda }}_\ell , \lambda _{T_\ell }^{S_1}, \lambda _{T_\ell }^{S_2}, \lambda _{T_\ell }^{S_3}) \in \{\eta ^{(1)}, \dots , \eta ^{(N)} \}^4$ where ${\underline{\lambda }}= \eta ^{(1)}< \dots < \eta ^{(N)}={\overline{\lambda }}$ and for the two types of elements ${{\hat{T}}} = {{\hat{T}}}^{(1)}$ and ${{\hat{T}}} = {{\hat{T}}}^{(2)}$, cf. Fig. 1. Since (58) has to be solved for $k=1,2$, this yields a total of $4 N^4$ finite element solutions of truncated transmission problems. In the online stage, given averaged values $({\varvec{\lambda }}_\ell , \lambda _{T_\ell }^{S_1}, \lambda _{T_\ell }^{S_2}, \lambda _{T_\ell }^{S_3}) \notin \{\eta ^{(1)}, \dots , \eta ^{(N)} \}^4$, the matrix ${\varvec{\Gamma }}_{{{\hat{T}}}}[({\varvec{\lambda }}_\ell , \lambda _{T_\ell }^{S_1}, \lambda _{T_\ell }^{S_2}, \lambda _{T_\ell }^{S_3})]$ is approximated by piecewise linear interpolation of the precomputed data.

In our experiments, in order to numerically approximate (58), we used the moderately large value $R=30$ for the radius of the computational domain and discretized it by a mesh consisting of about 4400 triangular elements and about 2300 vertices. We chose $N=16$ material points between ${\underline{\lambda }}= 1$ and ${\overline{\lambda }}= 1000$ which were chosen as stated in Table 1. Since increasing the number of material points *N* will drastically increase the precomputation time, the concrete choice of these points is of big importance. Thus, the points were chosen such that the interpolation error that is made in the online stage is as small as possible, see Remark 12. The total precomputation time for this setting was about two hours on a single core. Note that, for given PDE constraint, discretization method and material catalogue $\{\eta ^{(1)},\dots , \eta ^{(N)}\}$, this precomputation step has to be performed only once and can henceforth be used in all optimization runs.

#### Numerical Comparison of Models on Computational Domain

We make the same comparison of the two most promising models $\hat{{\mathcal {J}}}_{\text {SMWdiag}}$ (35) and $\hat{{\mathcal {J}}}_{\text {SMWapprox}}$ (60) as it was done for the homogeneous setting in Sect. 7.1.2. Again, recall that $\hat{{\mathcal {J}}}_{\text {TDnum}}$ coincides with $\hat{{\mathcal {J}}}_{\text {SMWapprox}}$ and is thus not examined separately.

**Fig. 15:**
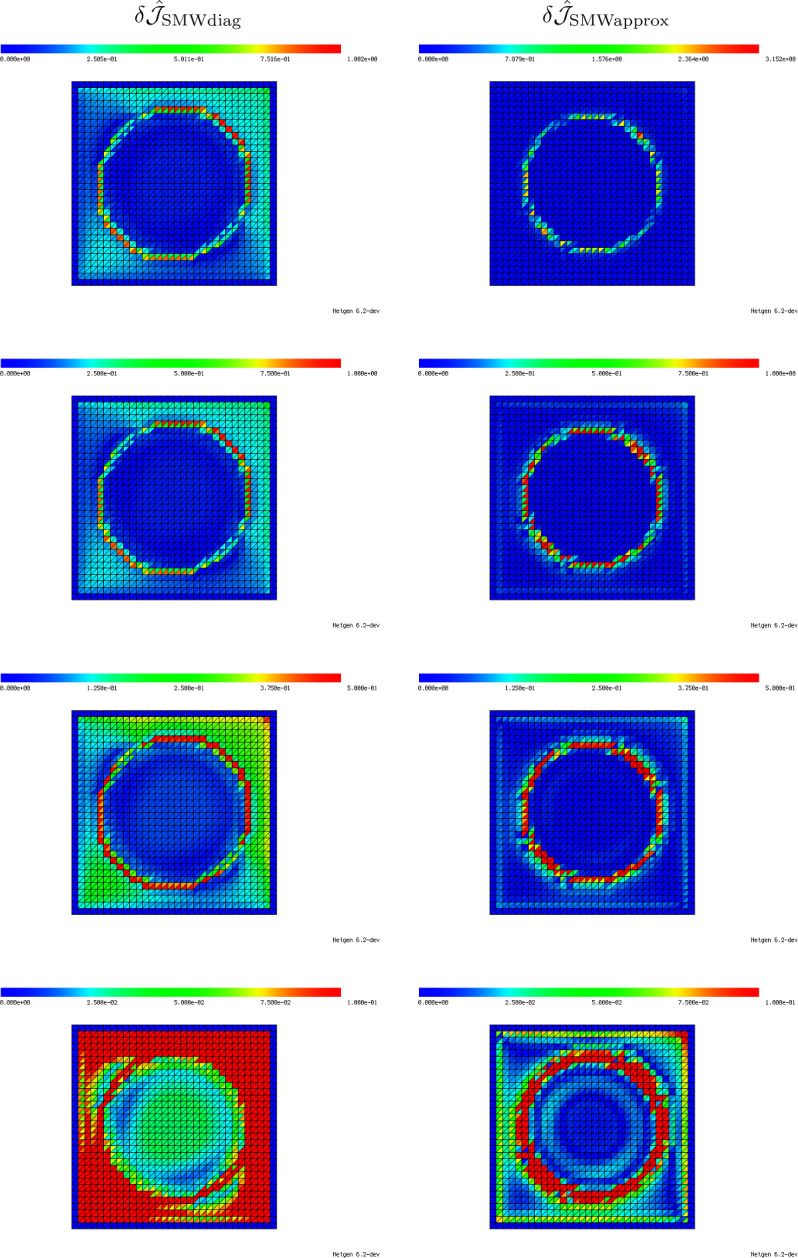
Comparison of relative errors $\delta \hat{{\mathcal {J}}}[T_\ell ]$ according to (72) for models $\hat{{\mathcal {J}}}_{\text {SMWdiag}}$ (35) in left column and $\hat{{\mathcal {J}}}_{\text {SMWapprox}}$ (60) ($=\hat{{\mathcal {J}}}_{\text {TDnum}}$ (52)) in right column for all interior elements $T_\ell$ in inhomogeneous setting. First line shows color plot according to their respective maximum errors. Second to fourth line show threshold for maximum relative error at $100 \%$, $50\%$ and $10\%$, respectively. Errors in elements touching the boundary are not computed

Figure 15 again shows the maximum relative error $\delta \hat{{\mathcal {J}}}$ of these two models over the computational domain. Again, different thresholds of the color bar are shown. As it was already observed in Sect. 7.1.2, the model $\hat{{\mathcal {J}}}_{\text {SMWapprox}}$ behaves particularly well in regions of homogeneous material. For both models, the largest errors occur at the transition from homogeneous material $\lambda (x) = {\underline{\lambda }}$ to inhomogeneous material.

**Fig. 16:**
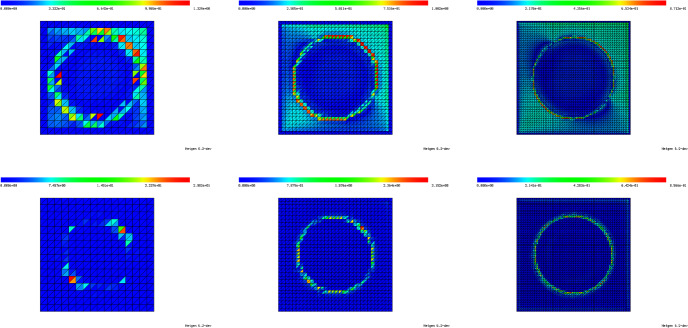
Comparison of relative errors in inhomogeneous setting for different mesh sizes for models $\hat{{\mathcal {J}}}_{\text {SMWdiag}}$ (top row) and $\hat{{\mathcal {J}}}_{\text {SMWapprox}} = \hat{{\mathcal {J}}}_{\text {TDnum}}$ (bottom row). Errors in elements touching the boundary are not computed

From Fig. 15 it can also be seen that the largest error of $\hat{{\mathcal {J}}}_{\text {SMWapprox}}$ is around $315\%$ compared to only about $100\%$ for $\hat{{\mathcal {J}}}_{\text {SMWdiag}}$. However, we mention that this effect disappears when a finer mesh is chosen as it is illustrated in Fig. 16. There, it can be seen that the maximum error of $\hat{{\mathcal {J}}}_{\text {SMWapprox}}$ in the refined mesh is only around $86\%$ which is in the same range as for $\hat{{\mathcal {J}}}_{\text {SMWdiag}}$. The reason for this improvement of $\hat{{\mathcal {J}}}_{\text {SMWapprox}}$ is that, for a given function $\lambda (x)$, as the mesh size decreases, the range of values to be averaged in the direct neighborhood of an element becomes smaller which results in a smaller error when computing the average values $\lambda _{T_\ell }^{S_k}$ (50), $k=1,2,3$. In general, the model $\hat{{\mathcal {J}}}_{\text {SMWapprox}}$ behaves well if material variations around a fixed element are small and makes larger approximation errors when large ranges of material values have to be averaged.

### Further Improvements

Finally, we point out several directions in which this research could be extended to further improve the models $\hat{{\mathcal {J}}}_{\text {SMWapprox}}$, $\hat{{\mathcal {J}}}_{\text {TDnum}}$.

#### Boundary Regions

**Fig. 17:**
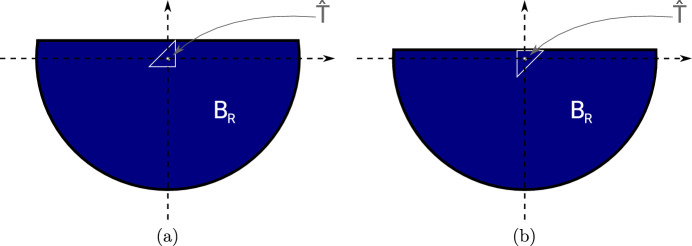
Setting for exterior problem (48) corresponding to elements **a** of type 1 or **b** of type 2 that touch a top boundary. On the circular part of the boundary of $B_R$, homogeneous Dirichlet conditions are set. The boundary conditions at top can be either of Dirichlet or Neumann type, depending on the physical problem

So far, we restricted our numerical results to regions away from the boundary and did not treat elements that touch the boundary. The reason for this is that, in the derivation of the models (52) and (60), the truncated exterior problems (48) and (58) are obtained by zooming in around the fixed element $T_\ell$ and rescaling. Thus, in the case where $T_\ell$ touches the boundary, the truncated domains depicted in Figs. 4 and 8 do not mimic the neighborhood of $T_\ell$. Instead, it would be more appropriate to perform precomputations on truncated half spaces as depicted in Fig. 17. The figure shows the setting of problem (57) in the case of a homogeneous material distribution corresponding to elements touching the top boundary. Also, here, inhomogeneous material can be treated by precomputing a range of combinations of material values in an offline stage and interpolating averaged sector values in the online stage. Here, the precomputation becomes a bit more involved since, in addition to accounting for different materials and different element types and $k=1,2$ in (57), one also has to distinguish between a left, bottom, right or top boundary as well as between Dirichlet or Neumann conditions imposed on that boundary. Thus, in order to also treat boundary regions of a rectangular domain ${\textsf{D}}$, an additional $32N^4$ truncated half space problems have to be solved in the offline stage. Here, *N* is the number of used material values, e.g., $N=16$.

**Fig. 18:**
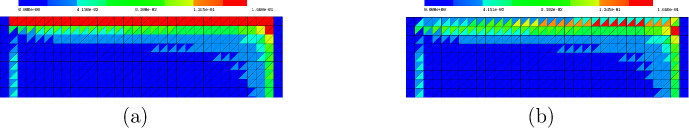
Comparison of model $\hat{{\mathcal {J}}}_{\text {SMWapprox}}$ in Neumann boundary region **a** without and **b** with special precomputation using truncated half spaces as depicted in Fig. 17. For comparison, the same color scale that is cut off at $16.6\%$ is used in (**a**) and (**b**). The maximum relative error in (**a**) is $77.89\%$ whereas it is only $16.6\%$ in (**b**)

In Fig. 18, we illustrate the improvement when elements touching the top boundary are given a special treatment by precomputing the matrix ${\varvec{\Gamma }}_{{{\hat{T}}}}[({\varvec{\lambda }}_\ell , \lambda _{T_\ell }^{S_1}, \lambda _{T_\ell }^{S_2}, \lambda _{T_\ell }^{S_3})]$ also for the reference elements ${{\hat{T}}}$ depicted in Fig. 17. In addition to the data presented in Fig. 15, we also computed the maximum relative errors in all elements touching the top boundary (of Neumann type). If the same data as in the interior is used, the maximum relative error is attained in the elements at the boundary and is as high as $77.89\%$. When the mentioned treatment of the boundary elements is used, the maximum error is still attained at an interior element and is only $16.6\%$.

#### Averaging of Inhomogeneous Material Distribution

**Fig. 19:**
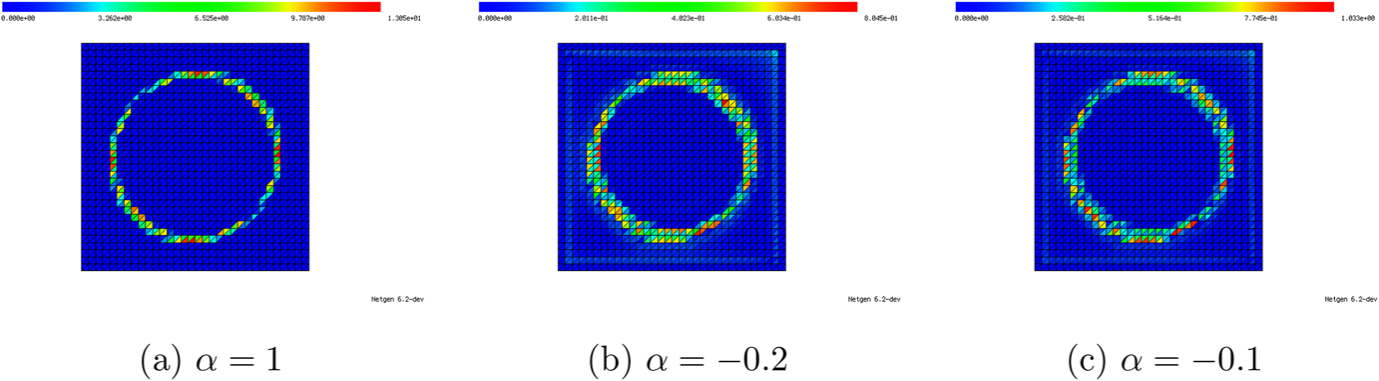
Maximum relative error $\delta \hat{{\mathcal {J}}}_{\text {SMWapprox}}$ over computational domain for different averaging parameters $\alpha$ in (50)

We mention that we observed that the way material values are averaged over sectors has a strong impact on the obtained relative error. Figure 19 shows the same experiments as discussed in Sect. 7.2 for different values of $\alpha$ in the averaging process (50). For $\alpha = 1$, the weighted Hölder mean (50) reduces to the weighted arithmetic mean which yields a large maximum error of about $1300\%$, see Fig. 19(a). Recall that our choice $\alpha = -0.5$ yielded the result in the right column of Fig. 15 with maximal value of $\delta \hat{{\mathcal {J}}} \approx 315\%$. Further numerical studies for $\alpha = -0.2$ and $\alpha = -0.1$ are depicted in Fig. 19b,c showing that for $\alpha = -0.2$ the maximal error in the mesh is actually smaller than for the model $\hat{{\mathcal {J}}}_{\text {SMWdiag}}$.

Moreover, one might want to think of decomposing the domain $B_R(0)$ into more than three sectors in order to reduce the error made by the averaging of inhomogeneous material parameters. However, here one should keep in mind that the number of truncated exterior problems to be solved in the precomputation stage with $n_{\text {sec}}$ sectors and *N* material points is of the order $N^{n_{\text {sec}}+1}$ and thus grows very fast with $n_{\text {sec}}$.

Finally, taking the average of material values on more than one layer of elements (cf. Fig. 6) together with suitable distance-dependent weights could lead to a better representation of the local material configuration and thus to potentially higher accuracy of the models $\hat{{\mathcal {J}}}_{\text {SMWdiag}}$ and $\hat{{\mathcal {J}}}_{\text {TDnum}}$ in an inhomogeneous setting.

## Conclusion and Outlook

In this paper, we introduced and examined different separable approximations to a discretized topology/material optimization problem. The Sherman–Morrison–Woodbury formula applied to the perturbed finite element stiffness matrix yielded a first separable exact model which, however, is prohibitively expensive to evaluate. A diagonal approximation of the stiffness matrix yielded a first tractable model. We introduced a model that is motivated by the continuous concept of topological derivatives for triangular inclusion shapes. Moreover, we also introduced a model that approximates the Sherman–Morrison–Woodbury model with high accuracy by performing similar rescaling steps as in the topological derivative model. Subsequently, we showed the somewhat surprising result that these latter two models coincide. Finally, we compared the performances of all models numerically. While the diagonal approximation of the Sherman–Morrison–Woodbury model can be evaluated very efficiently without any problems, the new models need data to be precomputed in an offline stage. In our model problem, however, we saw that the newly introduced models show significantly higher accuracy in most regions of the domain.

This work presented here can be extended and continued in several directions.

- We illustrated our methods for the case of the compliance functional in a stationary heat equation. We emphasize that this model was chosen for compactness of presentation and that extensions to other cost functions and other linear PDE constraints (e.g., linear elasticity) can be obtained in a rather straight-forward way (possibly yielding slightly more technical formulas). An extension to non-selfadjoint problems (including also nonlinear cost functions) could be realized taking into account Remark 6. An extension of models based on the Sherman–Morrison–Woodbury formula to other linear PDE constraints is straightforward since the structure of the discretized problem is the same as for our model problem. The topological derivative model can be extended to other PDE constraints following the general systematic procedure presented in [20].
- In this paper, we always assumed a structured mesh of a certain mesh topology to be given. While this is a common assumption made in many publications on topology optimization, an extension to general meshes with arbitrary element shapes and sizes would be an interesting topic of future research. In this setting, one might want to parametrize the shape of triangles. Then one could perform the precomputation for a (small) number of sample triangle shapes and interpolate their data in order to treat a family of element shapes.
- The extension of the proposed approaches to nonlinear PDE constraints such as nonlinear elasticity or nonlinear magnetostatics is another interesting yet challenging task. Also, here, the general procedure for obtaining topological derivatives [20] could be used to establish a model similar to $\hat{{\mathcal {J}}}_{\text {TDnum}}$.
- Finally, the ultimate goal of this research is to obtain good approximate sub-problems in an iterative optimization algorithm. While solving the actual optimization problem was beyond the scope of this paper and subject of future research, we mention that this can be carried out in a similar way to [19]. In particular, in [19] it was shown that a sequential global programming approach with a diagonal approximation of a Sherman–Morrison–Woodbury model was superior to the well-established method of moving asymptotes (MMA) [17] in terms of both number of optimization iterations and quality of obtained solutions. A similar or even better behavior is expected when replacing the diagonal approximation model to our model $\hat{{\mathcal {J}}}_{\text {SMWapprox}}$.

## Data Availability

The code and data used for all numerical experiments is available at https://zenodo.org/doi/10.5281/zenodo.11070440.
